# CD8+ T Lymphocytes in Pituitary Neuroendocrine Tumors: Friend or Foe?

**DOI:** 10.3390/cells15121115

**Published:** 2026-06-19

**Authors:** Valeria-Nicoleta Nastase, Amalia Raluca Ceausu, Iulia Florentina Burcea, Roxana Ioana Dumitriu-Stan, Pusa Nela Gaje, Flavia Zara, Marius Raica, Oana Albai, Catalina Poiana, Bogdan Timar

**Affiliations:** 1 Department of Microscopic Morphology/Histology, “Victor Babes” University of Medicine and Pharmacy, 300041 Timisoara, Romania; valeria.nastase@umft.ro (V.-N.N.);; 2 Angiogenesis Research Centre, “Victor Babes” University of Medicine and Pharmacy, 300041 Timisoara, Romania; 3 “C. I. Parhon” National Institute of Endocrinology, 011683 Bucharest, Romania; 4 Endocrinology Department, “Carol Davila” University of Medicine and Pharmacy, 020021 Bucharest, Romania; 5 Municipal Emergency Hospital, 300041 Timisoara, Romania; 6 Department of Second Internal Medicine-Diabetes, Nutrition, Metabolic Diseases, and Systemic Rheumatology, “Victor Babes” University of Medicine and Pharmacy, 300041 Timisoara, Romania; albai.oana@umft.ro (O.A.);; 7 Department of Diabetes, Nutrition and Metabolic Diseases Clinic, “Pius Brînzeu” Emergency Clinical County University Hospital, 300723 Timisoara, Romania; 8 Centre for Molecular Research in Nephrology and Vascular Disease/MOL-NEPHRO-VASC, “Victor Babes” University of Medicine and Pharmacy, 300041 Timisoara, Romania

**Keywords:** PitNETs, transcription factors, immune microenvironment, CD8+ lymphocytes

## Abstract

**Background:** The tumor immune microenvironment, particularly the role of cytotoxic CD8+ T lymphocytes, is crucial in cancer progression but remains poorly understood in pituitary neuroendocrine tumors (PitNETs). The significance of CD8+ cell infiltration varies across PitNET subtypes, suggesting a complex interplay with tumor cell lineage. This study aimed to characterize the distribution of CD8+ tumor-infiltrating lymphocytes across different PitNET subtypes defined by the current WHO classification and to explore their association with clinicopathological features. **Methods:** We conducted a retrospective study on 40 surgically resected PitNETs. All cases were classified based on immunohistochemical expression of pituitary hormones and lineage-specific transcription factors (PIT-1, TPIT, SF-1). CD8+ lymphocyte density was quantified using immunohistochemistry and calculated as cells/mm^2^. Exploratory statistical analysis was performed based on non-parametric tests to compare CD8+ cell density across tumor subtypes and with parameters like tumor size, invasiveness (Knosp grade), and proliferation index (Ki-67). Findings are to be treated as observational trends. **Results:** The highest density of CD8+ lymphocytes was observed in plurihormonal PIT-1-positive tumors [17.61 cells/mm^2^ (IQR: 17.61–60.36)], followed by somatotroph [13.2 (6.6–15.72)] and mammosomatotroph [13.83 (0–21.38)] tumors. A difference in CD8+ density was found between PIT-1-positive and PIT-1-negative tumors (n1 = 34, n2 = 6, U = 49.5, p_exact_ = 0.050, r = 0.33); the medium effect size indicates a possible lineage-related trend. Another difference was observed between SF-1-positive and SF-1-negative tumors (*p* = 0.025), with SF-1 lineage tumors showing the lowest infiltration. No correlations were found between CD8+ density and tumor size, Knosp grade, or Ki-67 index. **Conclusions:** The distribution of intratumoral CD8+ T lymphocytes in PitNETs is highly heterogeneous and appears to be strongly dictated by the transcription factor-defined tumor lineage rather than by traditional clinicopathological markers of aggressiveness. PIT-1 lineage tumors harbor a more active immune microenvironment, while SF-1 lineage tumors are relatively ‘immune-poor’. These findings highlight the immunological diversity of PitNETs and support further investigation of the tumor immune landscape. Collaborative multi-institutional studies are required to validate these trends.

## 1. Introduction

Pituitary neuroendocrine tumors (PitNETs) arise from the hormone-producing cells of the anterior pituitary gland, accounting for approximately 15% of all intracranial tumors. Epidemiological data indicate that the incidence of PitNETs has been increasing steadily over recent years, reflecting both improved diagnosis techniques and heightened clinical awareness [1,2]. Since 2017, their classification has been based on adenohypophyseal cell lineages, as defined by the expression of pituitary hormones and corresponding transcription factors, according to the World Health Organization (WHO) guidelines [3]. Although the majority of PitNETs exhibit indolent growth, and can be effectively managed through surgical or pharmacological interventions, certain cases demonstrates aggressive biological behavior, rendering their clinical management particularly challenging [4,5].

In this context, understanding the mechanisms involved in pituitary tumorigenesis is essential for the development of new targeted therapies. Studies on other neoplasms have demonstrated that, beyond the neoplastic cells themselves, the tumor microenvironment (TME) plays a crucial role in tumor growth, progression, and immune modulation [6]. The pituitary TME comprises extracellular matrix components, cytokines, vascular and lymphatic structures, fibroblasts, folliculo-stellate (FS) cells, and various immune cell populations that together form a dynamic network influencing tumor proliferation, angiogenesis, and immune regulation [7,8].

Tumor-associated macrophages (TAMs), dendritic cells, and tumor-infiltrating lymphocytes (TILs) form a major component of the immune cell compartment within solid tumors. These cells are actively involved in tumor antigen recognition and presentation, modulation of the local inflammatory milieu, and chemotactic recruitment of effector immune subsets. Through these processes, they influence the balance between immune-mediated tumor suppression and immune escape. Among the immune elements, T lymphocytes, particularly CD8+ cytotoxic T cells, play a pivotal role as direct effectors of antitumor immunity, mediating the recognition and destruction of neoplastic cells [9,10,11].

CD8+ cytotoxic T lymphocytes mediate the antitumor response by recognizing antigens presented on MHC class I molecules and inducing neoplastic cell death through mechanisms dependent on perforin, granzymes, and interferon-γ secretion. However, persistent antigenic stimulation can lead to functional exhaustion of these cells, characterized by decreased cytotoxic activity and expression of inhibitory receptors, such as PD-1, limiting the effectiveness of the antitumor immune response [12,13].

The precise biological significance of lymphocytic infiltration in PitNETs remains a central question as it may reflect either an active host antitumor immune response or, conversely, a state of ineffective immunity shaped by local immunosuppressive mechanisms and activation of immune checkpoint pathways. Moreover, the secretory activity of pituitary tumors directly influences the tumor immune microenvironment. Hormones such as growth hormone, prolactin, and glucocorticoids exert well-established immunomodulatory effects, potentially influencing both the recruitment and functional status of T lymphocytes within the tumor milieu [14]. Several immunohistochemical and transcriptomic studies have demonstrated the presence of cytotoxic T lymphocytes, with their density varying according to PitNET subtype. Notably, CD8+ lymphocyte infiltration has been reported primarily in functioning tumors, particularly in growth hormone-secreting lesions [9].

The concomitant identification of CD8+ T lymphocytes and immune checkpoint molecules in PitNETs suggests the existence of an active, rather than an inert, immune microenvironment. Recent immunohistochemical studies have revealed variable CD8+ lymphocyte expression and significant associations between lymphocytic infiltrate density, immune checkpoint activation, and clinicopathological parameters such as invasiveness, Knosp score, and tumor behavior. These observations support the hypothesis of a complex relationship between the local immune response and the biological aggressiveness of pituitary tumors [15,16].

Although the tumor immune microenvironment has been extensively investigated in numerous solid malignancies, data regarding PitNETs and other neuroendocrine tumors remain limited. In this context, a systematic evaluation of T lymphocyte distribution across tumor cell lineages defined by hormonal and transcription factor expression is warranted to better characterize the immunological heterogeneity of PitNETs. Therefore, the primary aim of this study was to systematically map the CD8+ T lymphocyte landscape across PitNET subtypes, anchored to the WHO classification of transcription factor lineages (PIT-1, TPIT, SF-1). A secondary objective was to determine whether this immune infiltration correlates with key clinicopathological parameters of tumor behavior, such as size, invasiveness, and proliferation.

## 2. Materials and Methods

The present study included 40 patients diagnosed with PitNETs based on clinical assessment, biochemical evaluation, and radiological imaging findings. The study protocol was approved by the Ethics Committee of the “Victor Babeș” University of Medicine and Pharmacy (Approval No. 102/03.10.2022, revised 2025). All patients underwent transsphenoidal surgical resection at one of the following institutions: the Neurosurgery Clinic of “Bagdasar-Arseni” Emergency Clinical Hospital (Bucharest, Romania), the Neurosurgery Clinic of “Colentina” Clinical Hospital (Bucharest, Romania), or the Neurosurgery Clinic of Brain Institute, Monza Hospital (Bucharest, Romania). Clinical and paraclinical variables collected for analysis included age at diagnosis, sex, maximum tumor diameter and Knosp grade, based on which tumors were categorized as non-invasive (grades 0–2), or invasive (grades 3–4).

The resected specimens were fixed in 10% neutral buffered formalin and processed according to standard histopathological protocols to obtain formalin-fixed, paraffin-embedded (FFPE) tissue blocks. These were subsequently subjected to morphological and immunohistochemical analyses at the Department of Histology and the Angiogenesis Research Center, “Victor Babeș” University of Medicine and Pharmacy, Romania. Histopathological diagnosis was established based on routine staining with hematoxylin and eosin (H&E) on 3 µm sections for each case. Morphological staining was performed using a Leica Auto-stainer XL (Leica Biosystem Newcastle Ltd., Balliol Business Park West, Benton Lane, New Castle Upon Tyne NE 12 EW, United Kingdom). Microscopic analysis was assessed with the Nikon Eclipse E 600 microscope (Nikon Corporation, Tokyo, Japan).

All PitNETs were classified according to the current WHO criteria, based on the immunohistochemical expression of anterior pituitary hormones and lineage-specific transcription factors. Hormonal differentiation was assessed based on cytoplasmic immunoreactivity (GH, PRL, ACTH, TSH, FSH, LH), while tumor cell lineage was established by nuclear expression of PIT-1, TPIT, and SF-1. Tumor cell proliferation, assessed by Ki-67 nuclear immunoreactivity, was evaluated semi-automatically using ImageJ (v. 2.0). The Ki-67 labeling index was determined in the areas showing the highest density of positively stained tumor nuclei (hot spots). Non-neoplastic elements, including stromal cells and vascular endothelial cells, were manually excluded from the analysis. The percentage of Ki-67-positive tumor nuclei was calculated relative to the total number of tumor nuclei within the selected hot-spot areas. A cutoff value of 3% was used for subsequent analyses. Tumor-infiltrating T lymphocytes were assessed by immunohistochemical staining for CD8 (membranous expression). For each case, the entire tumor section was initially evaluated at low magnification to identify areas with the highest density of CD8+ lymphocytes (hotspots). Subsequently, five intratumoral high-power fields (×400; objective ×40, ocular 10×/18) were selected from these hotspot regions for quantitative evaluation, while peripheral margins, necrotic areas, and non-neoplastic stromal regions were excluded. CD8-positive lymphocytes were manually counted in each field, and the arithmetic mean was calculated. To ensure objectivity, CD8-positive lymphocytes were counted independently by two pathologists who were blinded to the clinical and histopathological data. In case of significant discrepancy, the slides were re-evaluated jointly to reach a consensus. The results were subsequently converted from cells per high-power field to cells per mm^2^ for standardized reporting.

The primary antibodies are mentioned in Table 1. Bond Epitope Retrieval Solution 1 and 2, with pH values of 6 and 9 respectively, were used for unmasking (Leica Biosystems, Newcastle Ltd., Newcastle Upon Tyne NE 12 8EW, UK). Additionally, sections were treated with 3% hydrogen peroxide for 5 min to suppress endogenous peroxidase activity.

Statistical analysis was performed using Numiqo (Numiqo Team (2026). Numiqo: Online Statistics Calculator. Numiqo e.U. Graz, Austria. URL: https://numiqo.com) (Accessed on 1 March 2026). Frequencies of categorical variables (clinical and histopathological features, PitNET classification, and proliferation index status) were presented as absolute numbers, percentages, or both, where relevant. Continuous variables with a symmetric, normal distribution (age at diagnosis and maximum tumor diameter) were expressed as mean ± standard deviation, whereas heavily skewed data (density of CD8+ lymphocytes) were represented using the median and interquartile range (IQR) highlighting first and third quartiles (Q1, Q3). The distribution of continuous data was verified using quantile–quantile (Q–Q) plots to determine whether a normal distribution could be assumed. Given the small sample size, all statistical evaluations checking CD8+ lymphocyte density against clinical, radiological, and immunohistochemical parameters were treated as exploratory and hypothesis-generating. Raw unadjusted *p*-values are reported alongside absolute effect sizes (r) to mitigate the risk of Type II (false-negative) errors and preserve subtle biological signals. These findings are interpreted strictly as observational trends that establish a foundational baseline to guide future large-scale validation studies. Multi-subtype comparison was performed using the Kruskal–Wallis test, and comparison with age was done using Spearman’s Correlation while binary groups were evaluated using non-parametric Mann–Whitney U tests.

## 3. Results

### 3.1. Patient Characteristics

A total of 40 patients were included in the study. The cohort included 22 females (55%) and 18 males (45%), with a mean age at diagnosis of 47.8 ± 11.78 years. Based on biochemical assessment of hormonal secretion, 34 tumors (85%) were classified as functioning PitNETs, demonstrating active hormone hypersecretion at diagnosis, while 6 cases (15%) were identified as non-functioning.

The mean tumor diameter at diagnosis was 25.15 ± 11.52 mm. Using a 10 mm cutoff, 6 cases (15%) were classified as microadenomas, and 34 cases (85%) were considered macroadenomas. According to the Knosp grading system, 6 cases (15%) were classified as grade 0, 7 cases (17.5%) as grade 1, 11 cases (27.5%) as grade 2, 12 cases (30%) as grade 3, and 4 cases (10%) as grade 4. For analytical purposes, tumors with Knosp grades 0–2 were categorized as non-invasive, whereas those with grades 3–4 were considered invasive, consistent with cavernous sinus extension.

### 3.2. Histopathological Evaluation

**Figure 1:**
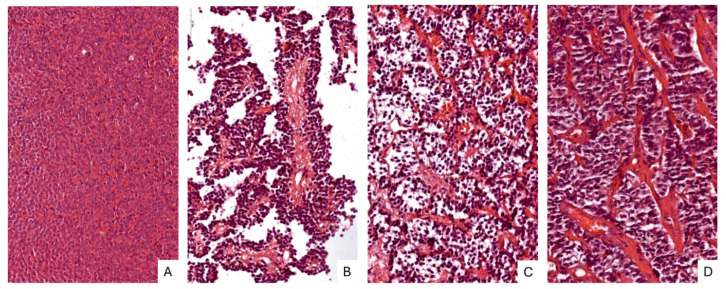
Tumoral growth pattern. (**A**) Acidophilic tumor exhibiting a diffuse architectural growth pattern; (**B**) papillary growth pattern with loose connective tissue with blood vessels in the center of the tumoral papilla; (**C**) chromophobic tumor cells arranged in an alveolar pattern; (**D**) trabecular growth pattern defined by thick connective tissue trabeculae separating tumor cell nests.

Histopathological analysis (using H&E staining) confirmed the definitive diagnosis (pituitary neuroendocrine tumor) and classified the tumors according to their growth pattern: 67.5% (*n* = 27) of cases presented a diffuse architectural pattern, and 17.5% (*n* = 7) were considered papillary tumors followed by 12.5% (*n* = 5) alveolar tumors; only one tumor was described as having a trabecular growth pattern (2.5%) (Figure 1).

### 3.3. Immunohistochemical Analysis

Immunohistochemical evaluation constituted a fundamental step in the accurate classification of PitNETs according to the current WHO criteria. Based on the evaluation of anterior pituitary hormones (GH, PRL, TSH, ACTH, FSH, LH) and lineage-specific transcription factors (PIT1, TPIT, SF1), the following subtypes were identified: 12 somatotroph tumors (immunopositive for GH and PIT-1), 11 mammosomatotroph tumors (immunopositive for GH, PRL, and PIT-1), 5 plurihormonal PIT-1-positive tumors (expressing GH, TSH, and/or PRL, PIT-1), and 6 gonadotroph tumors (immunopositive for FSH and/or LH, SF1). Additionally, 6 tumors were classified as unusual plurihormonal PitNETs, characterized by combined expression of multiple hormones and transcription factors. Within this latter category, three cases co-expressed GH and LH, two were positive for GH and ACTH, and one of the tumors presented positive immunoreaction for GH, TSH, LH.

The immunohistochemical findings for pituitary hormones and transcription factors are presented in Table 2.

**Figure 2:**
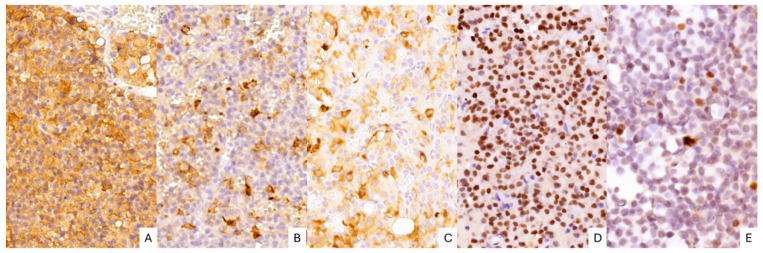
Plurihormonal PIT-1-positive PitNET with Ki-67 > 3% ((**A**)—GH cytoplasmic immunoreactivity; (**B**)—PRL cytoplasmic immunoreactivity; (**C**)—TSH cytoplasmic immunoreactivity; (**D**)—PIT1 nuclear immunoreactivity; (**E**)—Ki-67 nuclear immunoreactivity).

**Figure 3:**
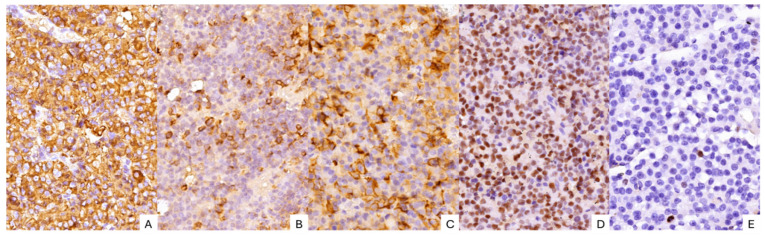
Plurihormonal PIT-1-positive PitNET with Ki-67 < 3% ((**A**)—GH cytoplasmic immunoreactivity; (**B**)—PRL cytoplasmic immunoreactivity; (**C**)—TSH cytoplasmic immunoreactivity; (**D**)—PIT1 nuclear immunoreactivity; (**E**)—Ki-67 nuclear immunoreactivity).

Evaluation of nuclear Ki-67 proliferation index expression revealed the proportion of actively dividing tumor cells. Based on previously reported thresholds, a cut-off value of 3% was used to stratify cases according to their proliferative potential. PitNETs with a Ki-67 index > 3%, indicative of potentially more aggressive biological behavior, were coded as 1 (Figure 2), whereas those with values < 3% were coded as 0 (Figure 3). An elevated Ki-67 index (>3%) was identified in five cases. The relationships between the proliferation index, tumor subtype, CD8+ lymphocyte density, and the clinical/paraclinical characteristics of the included subjects are summarized in Table 3.

**Figure 4:**
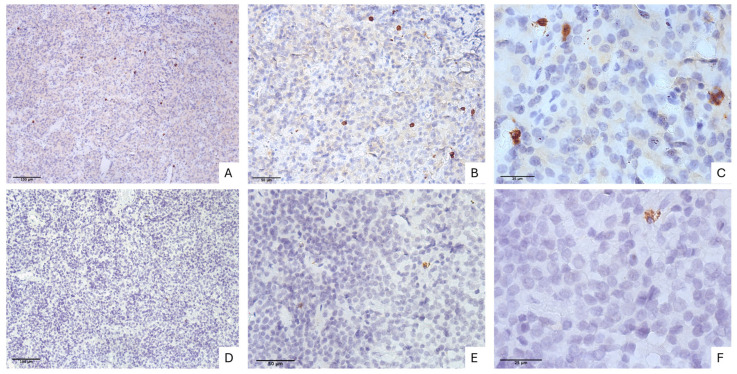
CD8+ lymphocytes distributed among tumor cells. Case 1—high density of positive cells: (**A**) low magnification, (**B**) high-power field, (**C**) cellular details. Case 2—low density of positive cells: (**D**) low magnification, (**E**) high-power field, (**F**) cellular details.

CD8-positive tumor-infiltrating lymphocytes (identified by predominantly membranous immunohistochemical expression with focal cytoplasmic enhancement) (Figure 4), as described in Section 2, were quantified across the analyzed cases. The median density of CD8+ lymphocytes [cells/mm^2^ (IQR: Q1–Q3)] was subsequently calculated for each tumor subtype.

**Figure 5:**
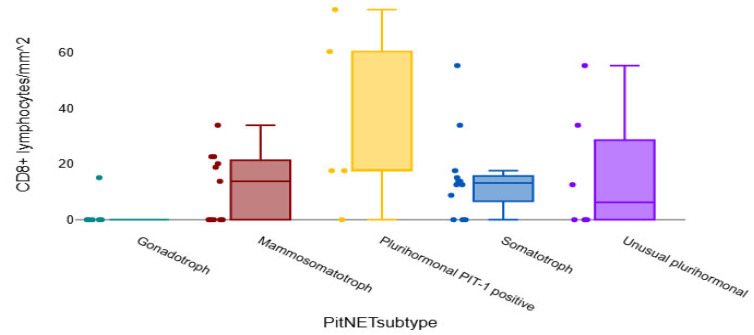
Intratumoral CD8+ lymphocyte density by PitNET subtype. Individual values are displayed as raw dots, the rectangular boxes represent the interquartile range (IQR; Q1 to Q3), the horizontal line inside each box marks the median value, and the whiskers represent the data range excluding outliers.

The highest density of CD8+ lymphocytes was observed in plurihormonal PIT1-positive PitNETs [17.61 cells/mm^2^ (IQR: 17.61–60.36)], followed by mammosomatotroph PitNETs [13.83 (0–21.38)], somatotroph PitNETs [13.2 (6.6–15.72)], and unusual plurihormonal PitNETs [6.29 (0–28.61)]. The lowest CD8+ lymphocyte density was identified in gonadotroph PitNETs [0 (0–0)]. Notably, substantial variability was observed within several subgroups (Figure 5).

The clinico-paraclinical characteristics of the patients, and the mean CD8+ lymphocyte density across tumor subtypes, are mentioned in Table 3.

Among the 12 somatotroph PitNETs included in current study, intratumoral CD8+ lymphocytes were identified in 9 cases, with a maximum density of 55.3 cells/mm^2^. Of the 11 mammosomatotroph PitNETs, CD8+ lymphocytes were observed in 6 cases, reaching a maximum density of 34 cells/mm^2^. Within plurihormonal PIT1-positive PitNETs (*n* = 5), intratumoral lymphocytes were detected in 4 cases, with the highest density recorded in this subgroup (60.4 cells/mm^2^). In the subgroup of gonadotroph PitNETs, intratumoral CD8+ lymphocytes were visualized in only one case, with a density of 15 cells/mm^2^. In tumors with unusual hormonal associations (*n* = 6), intratumoral lymphocytes were present in three cases, with densities ranging from 12 to 55 cells/mm^2^.

A statistical evaluation across all 5 PitNET subtypes revealed no overall statistically significant difference in intratumoral CD8+ lymphocyte density (Kruskal–Wallis test, H = 6.49, *p* = 0.166). No further statistical testing was performed on the subtype pairs.

Higher intratumoral CD8+ lymphocyte densities (cells/mm^2^) were observed in PIT1-positive and GH-positive PitNETs compared with tumors lacking PIT1 expression (*p* = 0.05). Within the same PIT1-positive subgroup, tumors without PRL IHC expression displayed a lower median CD8+ lymphocyte density (cells/mm^2^) (Mdn = 12.60) compared with tumors with positive PRL expression (Mdn = 17.60); however, this difference was not statistically significant (*p* = 0.945).

In contrast to PIT1-positive PitNETs, SF1-positive tumors presented lower CD8+ lymphocyte densities (cells/mm^2^) compared with tumors without SF1 expression (*p* = 0.025).

According to hormonal secretory status, intratumoral CD8+ lymphocytes were identified in 22 of 34 functional PitNETs (64.7%). In contrast, among the six non-functioning tumors, defined by the absence of detectable hormone hypersecretion, only one case exhibited CD8+ lymphocytes within the tumor.

**Figure 6:**
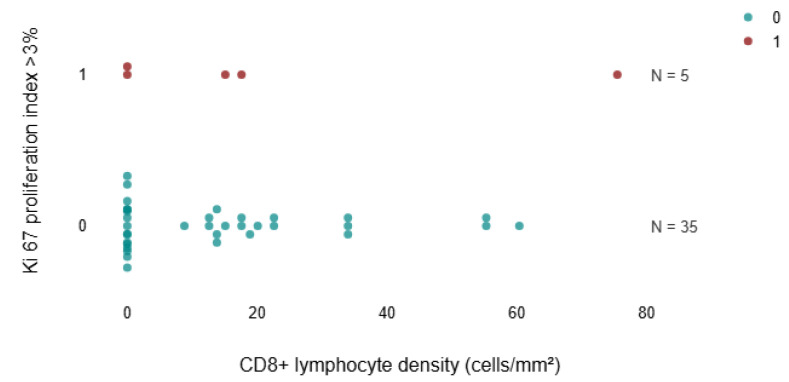
Distribution of CD8+ lymphocyte densities across Ki-67 proliferation groups.

No significant associations were observed between intratumoral CD8+ lymphocyte density and clinical features such as patient sex (*p* = 0.052), age at diagnosis (Spearman’s r = −0.08, *p* = 0.636), micro/macroadenoma classification (*p* = 0.649), or invasiveness indicators such as Knosp grade (0–2, 3–4) (*p* = 0.597) or Ki-67 (*p* = 0.467) which can be observed in Figure 6.

### 3.4. Statistical Analysis

**Figure 7:**
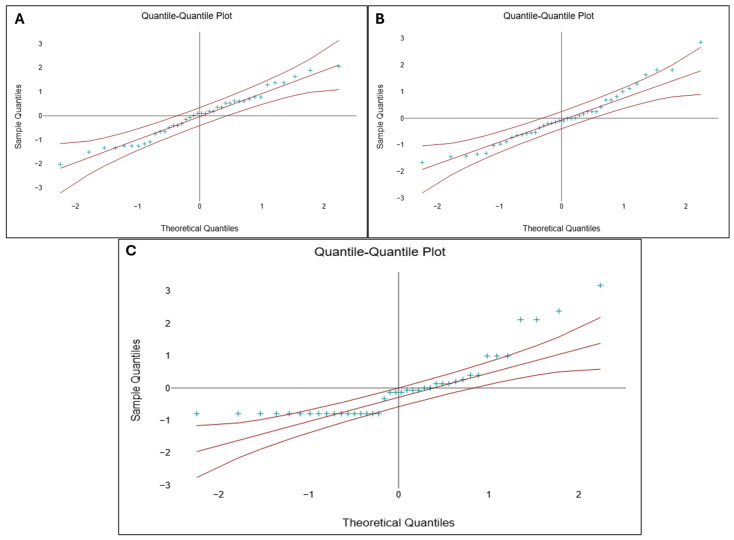
Q–Q plots for normal distribution analysis of primary clinical and pathological parameters; (**A**) Age at diagnostic; (**B**) Maximum tumor diameter; (**C**) CD8+ lymphocytes/mm^2^.

Assessment of data normality revealed that patient age at diagnosis and maximum tumor diameter followed a normal distribution. In contrast, CD8+ lymphocytes/mm^2^ exhibited significant non-normality; the Q-Q plots demonstrated a pronounced right-skewness and a distinct floor effect at the lower quantiles, with several observations falling outside the 95% confidence intervals (Figure 7). Because CD8+ lymphocytes/mm^2^ were not normally distributed, non-parametric tests were used for statistical analysis.

To limit the risk of Type I (false-positive) errors due to the small sample size (*n* = 40), all statistical evaluations checking CD8+ lymphocyte density against clinical, radiological, and immunohistochemical parameters were considered as exploratory and hypothesis-generating. The *p*-values were reported raw and unadjusted together with the absolute effect sizes (r) to mitigate the risk of Type II (false-negative) errors. These findings are to be treated as observational trends which need to be validated by large-scale validation studies.

The Kruskal–Wallis test was run for PitNET subtypes to check for differences in intratumoral CD8+ lymphocyte density across the 5 subtypes. There was no statistically significant difference (H = 6.49, *p* = 0.166). Statistical testing on the subtype pairs was not performed.

A Mann–Whitney U test revealed a statistically significant difference in CD8+ lymphocyte density (cells/mm^2^) between PIT1-positive and PIT1-negative adenomas (U = 49.50, n1 = 6, n2 = 34, *p* = 0.05, r = 0.33). The calculated effect size indicated a moderate magnitude of the difference. The same test was subsequently applied to the PIT1-positive subgroup (*n* = 34) and did not demonstrate a statistically significant difference in CD8+ lymphocyte density between PRL-positive and PRL-negative tumors within this lineage (U = 140.00, n1 = 19, n2 = 15 *p* = 0.945).

A Mann–Whitney U test also demonstrated a statistically significant difference in CD8+ lymphocyte density (cells/mm^2^) between SF1-positive adenomas and tumors without SF1 expression (U = 77.50, n1 = 30, n2 = 10 *p* = 0.025, r = 0.37). The calculated effect size indicated a moderate magnitude of the difference.

To screen for other clinical and histopathological features, parallel exploratory tests were conducted.

The result of the Spearman correlation showed that there was a negligible, negative correlation between age at diagnosis and CD8+ lymphocyte density (cells/mm^2^) (r(38) = −0.08, *p* = 0.636).

Mann–Whitney U tests were applied for features such as patient sex (*p* = 0.052), micro/macroadenoma classification (*p* = 0.649), or invasiveness indicators such as Ki-67 (*p* = 0.467) or Knosp grade (0–2, 3–4) (*p* = 0.597).

## 4. Discussion

The principal finding of this study is that the density of intratumoral CD8+ T lymphocytes in PitNETs appears to vary according to transcription factor-defined tumor lineage. Specifically, our data suggest that PIT-1 lineage tumors, particularly plurihormonal variants, may harbor higher CD8+ infiltrates, whereas SF-1 lineage tumors tend to display a relatively immune-poor microenvironment. These observations should be interpreted cautiously, given the exploratory design, the limited sample size, and the absence of statistically significant differences across all five subtypes in the global comparison.

The current findings suggest an important heterogeneity of intratumoral CD8+ lymphocyte infiltration across PitNET subtypes, suggesting the complex and dynamic nature of the tumor immune microenvironment. This variability is consistent with previous reports indicating that immune cell distribution in PitNETs is not uniform, but rather influenced by tumor cell lineage, hormonal activity, and biological behavior [17,18]. In this context, tumor subtype, as defined by transcription factor-driven lineage and associated hormonal expression, appears to play a central role in shaping the composition and density of intratumoral lymphocytic infiltration [19].

Consistent with these observations, our findings reflect that PIT1-lineage PitNETs displayed greater intratumoral CD8+ lymphocyte densities compared to other tumor subtypes, with plurihormonal PIT1-positive tumors showing the highest levels.

Recent transcriptomic and single-cell studies have revealed that PIT1-lineage tumors contain a more active immune microenvironment compared with other PitNET subtypes. For instance, single-cell RNA sequencing analyses have shown that PIT1-driven tumors exhibit increased infiltration of T lymphocytes, including CD8+ cells, whereas SF1-lineage tumors present a relatively immune-poor phenotype [20,21]. Similarly, comprehensive reviews of the PitNET immune landscape have reported that the cellular composition of the tumor microenvironment varies significantly across transcription factor-defined lineages, with PIT1-positive tumors being characterized by a higher density of intratumoral lymphocytes [22].

Previous studies have reported more increased densities of tumor-infiltrating lymphocytes, including CD8+ T cells, in GH-secreting PitNETs compared with other subtypes [23]. This aspect may be explained by the immunomodulatory effects of growth hormone and IGF1, which have been shown to influence cytokine production, including IL-6 and TNF-α, but also interferon signaling pathways, thereby contributing to a more active tumor immune microenvironment. This pattern is consistent with our results, somatotroph tumors presenting relatively elevated CD8+ lymphocyte densities [24,25,26].

Although hormonal activity modulates the immune landscape, the effects of specific hormones appear to be highly heterogeneous. In our cohort, no statistically significant differences in CD8+ lymphocyte density were observed between PRL-positive and PRL-negative tumors within the PIT1-positive subgroup. Previous studies have demonstrated that prolactin (PRL) exerts immunomodulatory effects through its receptor, which is expressed on diverse immune cell populations, influencing lymphocyte activation, proliferation, and survival. However, most of these effects have been described at the systemic level and do not necessarily translate into increased lymphocyte recruitment within the tumor. The accumulation of CD8+ T lymphocytes in the tumor microenvironment is primarily regulated by local factors, including chemokine gradients (e.g., CXCL9, CXCL10, CXCL11, and CCL5), endothelial adhesion mechanisms, and the overall inflammatory context of the tumor. Consequently, PRL expression, by itself, may be insufficient to promote CD8+ cell infiltration in the absence of a permissive chemokine microenvironment. Furthermore, PRL immunoreactivity does not necessarily reflect activation of prolactin-dependent signaling pathways in the tumor microenvironment, as prolactin receptor expression and downstream signaling pathway activity were not assessed in the present study. Another possible explanation is that immune cell recruitment in PIT-1 tumors is predominantly driven by lineage-specific molecular and inflammatory programs rather than by the secretion of a single pituitary hormone. Thus, the characteristic inflammatory phenotype previously described for PIT-1 tumors may have a greater influence on the immune infiltrate than the specific contribution of PRL expression. Finally, the pleiotropic and context-dependent nature of prolactin signaling, together with the relatively small sample size, may have further contributed to the lack of a detectable association between PRL expression and CD8+ lymphocyte infiltration observed in our study [27,28,29,30,31].

In contrast to PIT1-lineage tumors, SF1-positive PitNETs presented significantly lower intratumoral CD8+ lymphocyte densities in our study. This finding is consistent with previous molecular and transcriptomic studies suggesting that SF1-lineage tumors exhibit a relatively immune-depleted microenvironment compared with other PitNET subtypes. One potential explanation is the existence of lineage-specific differences in chemokine and cytokine expression, as recent transcriptomic analyses have demonstrated distinct immune-related gene signatures among PIT1-, TPIT-, and SF1-lineage tumors. Since CD8+ T-cell recruitment largely depends on chemokine-mediated mechanisms, reduced expression of T-cell-attracting chemokines may contribute to the limited lymphocytic infiltration observed in SF1-positive tumors. Furthermore, SF1-lineage tumors are generally characterized by a less pronounced inflammatory phenotype and may generate weaker immune stimulation than PIT1-lineage tumors [15,20,32,33].

An additional observation in our study is the important variability in CD8+ lymphocyte density across tumor subtypes, as seen in the box plot in Figure 5, supporting the concept of a highly heterogeneous tumor immune microenvironment in PitNETs, not only between different subtypes but also within the same tumor lineage. This aspect underscore the complex interplay between tumor-intrinsic factors, hormonal influences and local immune response [34,35].

Moreover, our results showed a higher prevalence of intratumoral CD8+ lymphocytes in functioning PitNETs compared with non-functioning tumors, in which lymphocytic infiltration was only rarely observed. Similar findings have been reported in previous studies, further supporting the existence of a complex interplay between endocrine and immune mechanisms within the tumor microenvironment (TME) and suggesting that endocrine activity may contribute to shaping the immune landscape of these tumors [16]. Several anterior pituitary hormones, particularly GH and PRL, possess immunomodulatory properties and may influence lymphocyte activation, proliferation, survival, and cytokine production through receptors expressed on various immune-cell populations. However, these effects have been predominantly described at the systemic level and are unlikely to directly account for intratumoral lymphocyte accumulation. As discussed above, immune-cell recruitment is governed by a complex network of local factors, including chemokine gradients, cytokine signaling and lineage-specific molecular programs. Therefore, hormonal activity should be considered one component of a multifactorial process rather than the primary determinant of immune infiltration. Nevertheless, endocrine hyperfunction is frequently accompanied by systemic metabolic and inflammatory alterations that may indirectly affect tumor–immune interactions and contribute to differences in the composition of immune-cell populations within the TME [30,36,37,38].

In contrast, non-functioning PitNETs are predominantly represented by gonadotroph (SF1-lineage) tumors, a subtype that has been associated with a relatively immune-depleted TME (as we already mentioned). In addition, the absence of clinically significant hormonal hypersecretion may limit endocrine–immune interactions that could otherwise contribute to immune-cell activation and modulation. Taken together, these factors may account for the reduced density of CD8+ lymphocytes observed in non-functioning tumors in our cohort and suggest that both lineage-specific molecular characteristics and the lack of overt endocrine activity contribute to a less permissive environment for the recruitment of cytotoxic T lymphocytes within the TME [15,39].

In our study, no significant associations were observed between intratumoral CD8+ lymphocyte density and clinicopathological parameters, including patient sex, age at diagnosis, tumor size, or Knosp grade. However, previous studies have suggested that patient characteristics, such as age, may influence the intratumoral immune landscape. In particular, younger individuals have been reported to exhibit reduced lymphocytic infiltration in certain tumor types [40]. Moreover, these findings are also supported by experimental models, where younger mice presented decreased T-cell infiltration in melanoma [41].

Regarding tumor aggressiveness, the relationship between CD8 + lymphocytic infiltration and invasive behavior in PitNETs remains incompletely understood. The majority of studies have mentioned that more aggressive tumors (defined by higher Knosp grades, increased risk of recurrence, resistance to first-line therapies) tend to exhibit lower CD8+ lymphocyte infiltration, reflecting a less active antitumoral immune response [9,42]. This concept is further supported by findings in other tumor types, for example in gastric cancer [43]. In contrast, other reports have described an opposite pattern, which describes increased lymphocytic infiltration in more invasive PitNETs [44]. Altogether, these observations reflect the complex and highly variable role of the immune microenvironment in determining tumor aggressiveness.

Although the current study did not specifically investigate immune checkpoint molecules, the observed correlations and variability in CD8+ lymphocyte distribution suggest that immune-related mechanisms may contribute to shaping tumor behavior. In this context, increasing attention has been directed toward the potential application of immunotherapy in aggressive and standard treatment-resistant pituitary tumors, in order to modulate the immune response and achieve an improved disease control. Accordingly, the assessment of immune system components may provide valuable insights into tumor biology, new prognosis factors, while also facilitating the identification of novel therapeutic strategies, particularly in tumors refractory to standard treatments [45,46,47].

This study has several limitations. Its retrospective design may introduce inherent biases. The relatively small sample size, particularly after stratification into five tumor subtypes according to the WHO classification, resulted in a limited number of cases within each subgroup, which may affect the statistical power and generalizability of the findings. In addition, the study was conducted in a single center, potentially limiting the external validity of the results.

## 5. Conclusions

In conclusion, our findings suggest that the CD8+ T-cell landscape in PitNETs may differ according to transcription factor-defined tumor lineage. In particular, PIT-1 lineage tumors appeared to show higher levels of CD8+ infiltration, whereas SF-1 lineage tumors tended to exhibit lower immune-cell density. Because these analyses were exploratory and based on a relatively small cohort, these observations should be interpreted as hypothesis-generating rather than definitive. Future larger, multi-institutional studies are needed to validate these patterns, clarify the functional status of infiltrating lymphocytes, and determine their potential prognostic or therapeutic relevance.

## Figures and Tables

**Table 1:** Antibodies used for anterior pituitary hormones, transcription factors, proliferation index and CD8+ T lymphocytes.

	Expression Pattern	Company	Clone	Dilution Factor
GH	Cytoplasmic	Dako Agilent	Polyclonal rabbit anti-human	1:400
PRL	Cytoplasmic	Dako Agilent	Polyclonal rabbit anti-human	1:300
TSH	Cytoplasmic	Thermo Fisher Scientific	TSH01 + TSH02	1:400
ACTH	Cytoplasmic	Dako Agilent	C93	1:50
FSH	Cytoplasmic	Thermo Fisher Scientific	FSH03	1:500
LH	Cytoplasmic	Thermo Fisher Scientific	LH01	1:500
PIT1	Nuclear	Thermo Fisher Scientific	Rabbit polyclonal antibody	1:500
Anti-Tpit	Nuclear	Abcam	CL6251	1:1000
SF1	Nuclear	Invitrogen (Thermo Fisher Scientific)	SF1 antibody PA5-79984	1:500
Ki-67	Nuclear	Thermo Fisher Scientific	MM1, RTU	RTU
CD8	Membranous	Leica Bond	4B11	RTU

GH—growth hormone, PRL—prolactin, TSH—thyroid-stimulating hormone, ACTH—adrenocorticotropic hormone, FSH—follicle-stimulating hormone, LH—luteinizing hormone, PIT1—pituitary-specific transcription factor, TPIT—T-box transcription factor, SF1—steroidogenic factor 1, CD8—cluster of differentiation 8; Dako, Agilent, Santa Clara, CA, USA; Thermo Fisher Scientific, Waltham, MA, USA; Abcam, Waltham, MA, USA; Leica Bond, Leica Biosystems, Buffalo Grove, IL, USA.

**Table 2:** PitNETs subtypes.

	GH	PRL	TSH	ACTH	FSH	LH	PIT1	TPIT	SF1
Somatotroph (*n* = 12)	+ (*n* = 12)	-	-	-	-	-	+ (*n* = 12)	-	-
Mammosomatotroph (*n* = 11)	+ (*n* = 11)	+ (*n* = 11)	-	-	-	-	+ (*n* = 11)	-	-
Plurihormonal PIT-1 positive (*n* = 5)	+ (*n* = 5)	+ (*n* = 4)	+ (*n* = 5)	-	-	-	+ (*n* = 5)	-	-
Gonadotroph (*n* = 6)	-	-	-	-	+ (*n* = 2)	+ (*n* = 3)	-	-	+ (*n* = 6)
Unusual Plurihormonal (*n* = 6)	+ (*n* = 6)	-	+ (*n* = 1)	+ (*n* = 2)	-	+ (*n* = 4)	+ (*n* = 6)	+ (*n* = 2)	+ (*n* = 4)

GH—growth hormone, PRL—prolactin, TSH—thyroid-stimulating hormone, ACTH—adrenocorticotropic hormone, FSH—follicle-stimulating hormone, LH—luteinizing hormone, PIT1—pituitary-specific transcription factor, TPIT—T-box transcription factor, SF1—steroidogenic factor 1, *n*—number of cases.

**Table 3:** Clinico-paraclinical characteristics and CD8+ lymphocyte density in PitNET subtypes.

PitNETsubtype	Gonadotroph	Mammosomatotroph	Somatotroph	Plurihormonal PIT-1 Positive	Unusual Plurihormonal
Frequency N (%)	6 (15%)	11 (27.5%)	12 (30%)	5 (12.5%)	6 (15%)
Male	1 (2.5%)	9 (22.5%)	6 (15%)	4 (10%)	2 (5%)
Female	5 (12.5%)	2 (5%)	6 (15%)	1 (2.5%)	4 (10%)
Age at diagnosis Mean ± Std.	50.5 ± 4.32	48.73 ± 13.6	44.33 ± 12.96	52.4 ± 8.53	46.5 ± 14.27
Maximum tumor diameter Mean ± Std.	39.67 ± 11.84	23.07 ± 12.82	21.85 ± 7.25	19.2 ± 5.09	26.02 ± 10.21
Functioning PitNETs N (%)	0 (0%)	11 (27.5%)	12 (30%)	5 (12.5%)	6 (15%)
Non-functioning PitNETs N (%)	6 (15%)	0 (0%)	0 (0%)	0 (0%)	0 (0%)
Macroadenoma N (%)	6 (15%)	7 (17.5%)	11 (27.5%)	4 (10%)	6 (15%)
Microadenoma N (%)	0 (0%)	4 (10%)	1 (2.5%)	1 (2.5%)	0 (0%)
Knosp Grade Score 0–2 non-invasive N (%).	0 (0%)	7 (17.5%)	10 (25%)	3 (7.5%)	4 (10%)
Knosp Grade Score 3–4 invasive N (%).	6 (15%)	4 (10%)	2 (5%)	2 (5%)	2 (5%)
Ki 67 proliferation index < 3% N (%)	5 (12.5%)	10 (25%)	11 (27.5%)	3 (7.5%)	6 (15%)
Ki 67 proliferation index > 3% N (%)	1 (2.5%)	1 (2.5%)	1 (2.5%)	2 (5%)	0 (0%)
CD8+ lymphocytes/mm^2^	0 (0–0)	13.83 (0–21.38)	13.2 (6.6–15.72)	17.61 (17.61–60.36)	6.29 (0–28.61)

## Data Availability

Data is contained within the article and Supplementary Material.

## Supplementary Materials

The following supporting information can be downloaded at: https://www.mdpi.com/article/10.3390/cells15121115/s1, Figures S1–S4: The original pathology images.
