# Supplementary figures and images for: CD8+ T Lymphocytes in Pituitary Neuroendocrine Tumors: Friend or Foe?
Source: Cells. 2026 Jun 19;15(12):1115. doi: 10.3390/cells15121115 (PMC13297189; doi:10.3390/cells15121115)

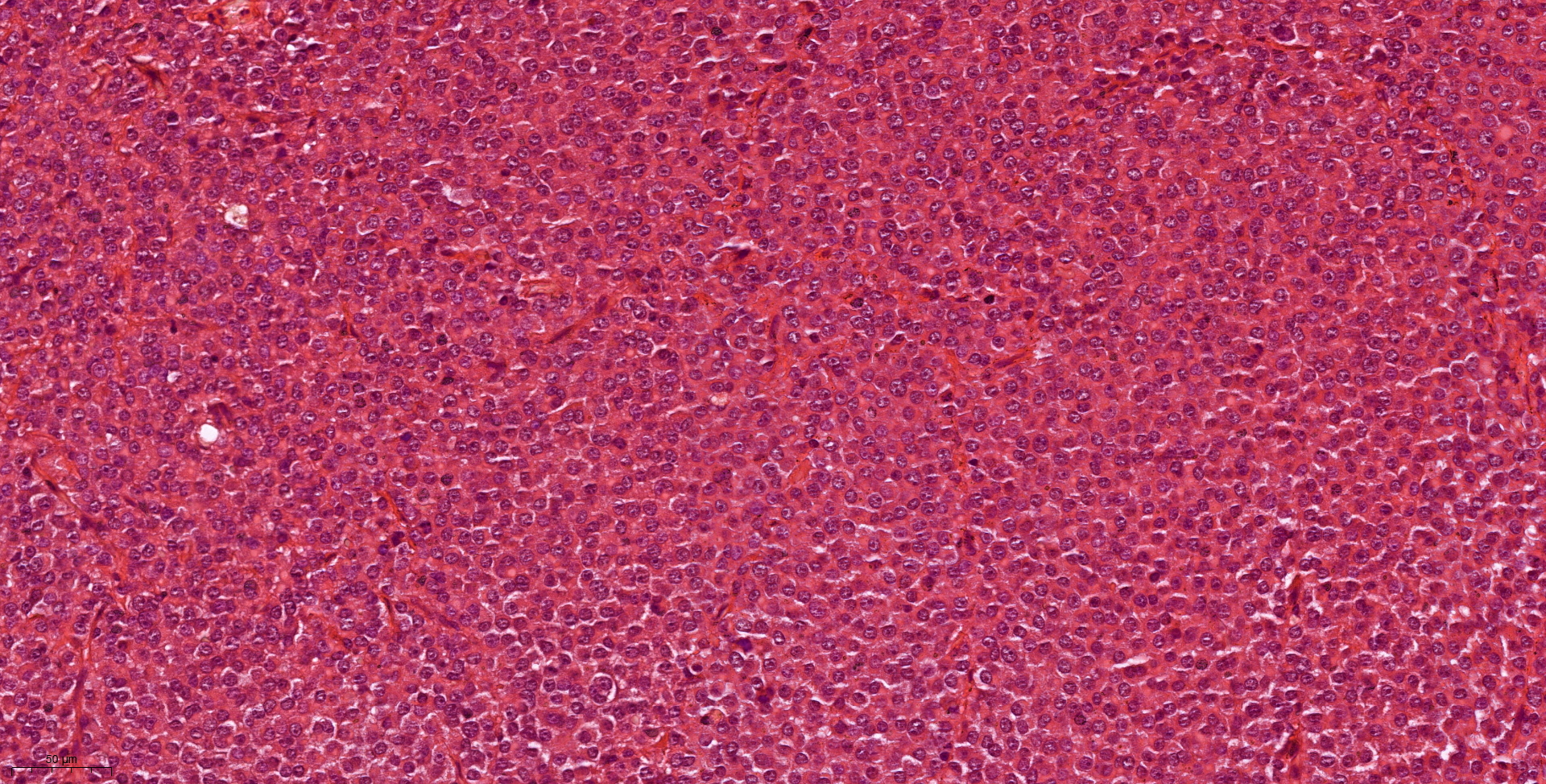

Supplement: Supplementary file 1 [file cells-15-01115-s001.zip › Supplementary Material/Figure1A.jpg]

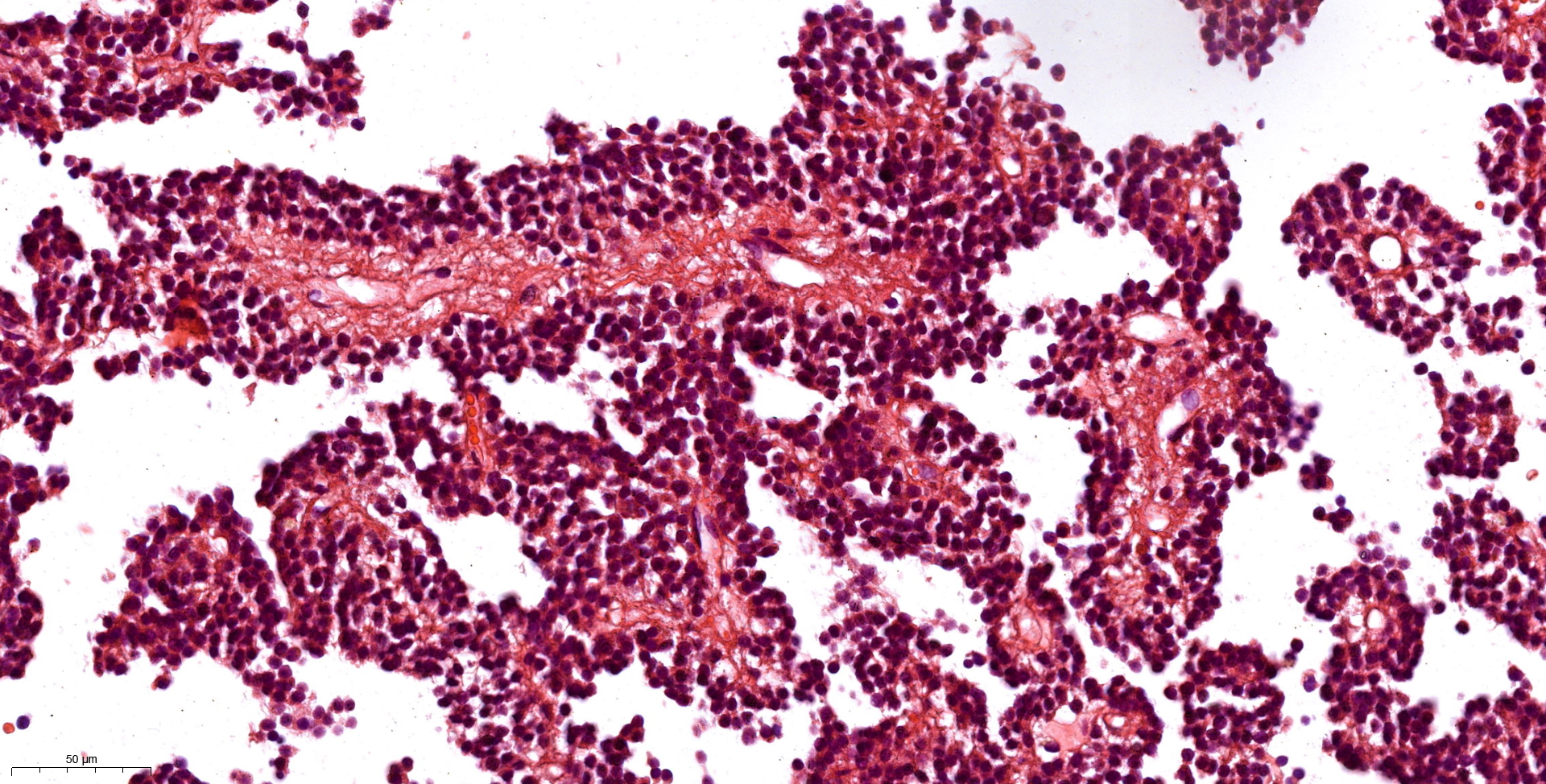

Supplement: Supplementary file 1 [file cells-15-01115-s001.zip › Supplementary Material/Figure1B.jpg]

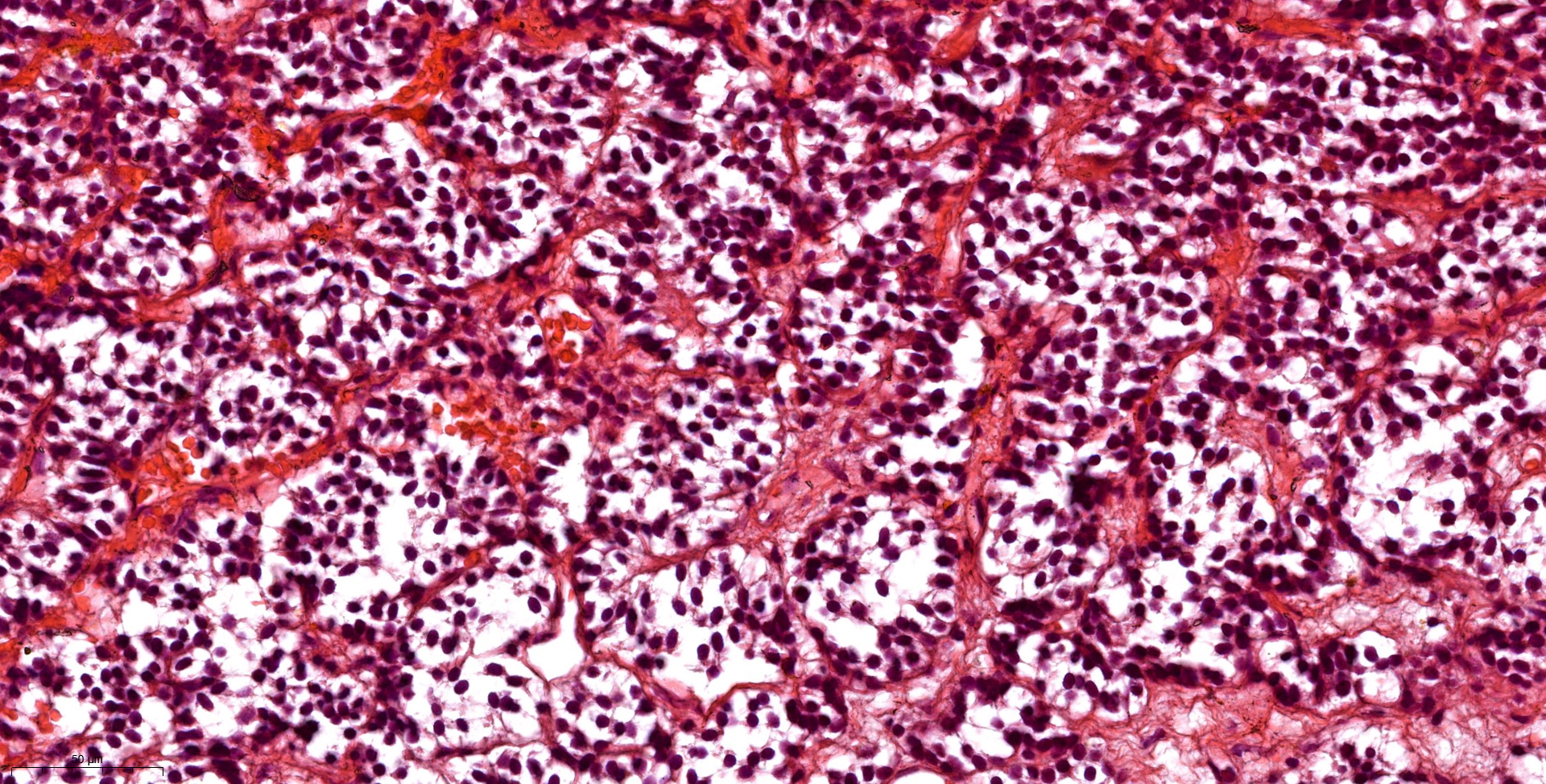

Supplement: Supplementary file 1 [file cells-15-01115-s001.zip › Supplementary Material/Figure1C.jpg]

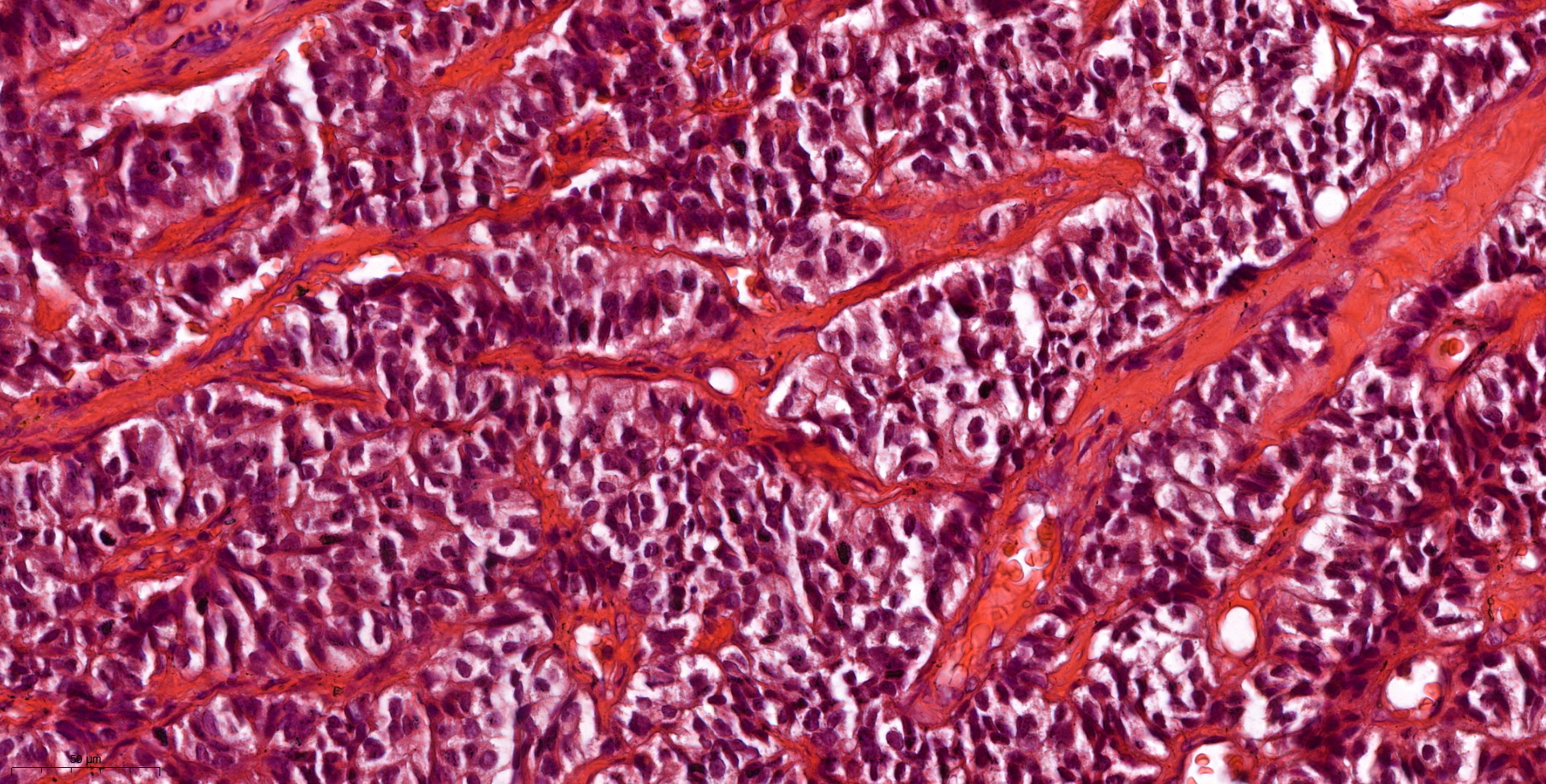

Supplement: Supplementary file 1 [file cells-15-01115-s001.zip › Supplementary Material/Figure1D.jpg]

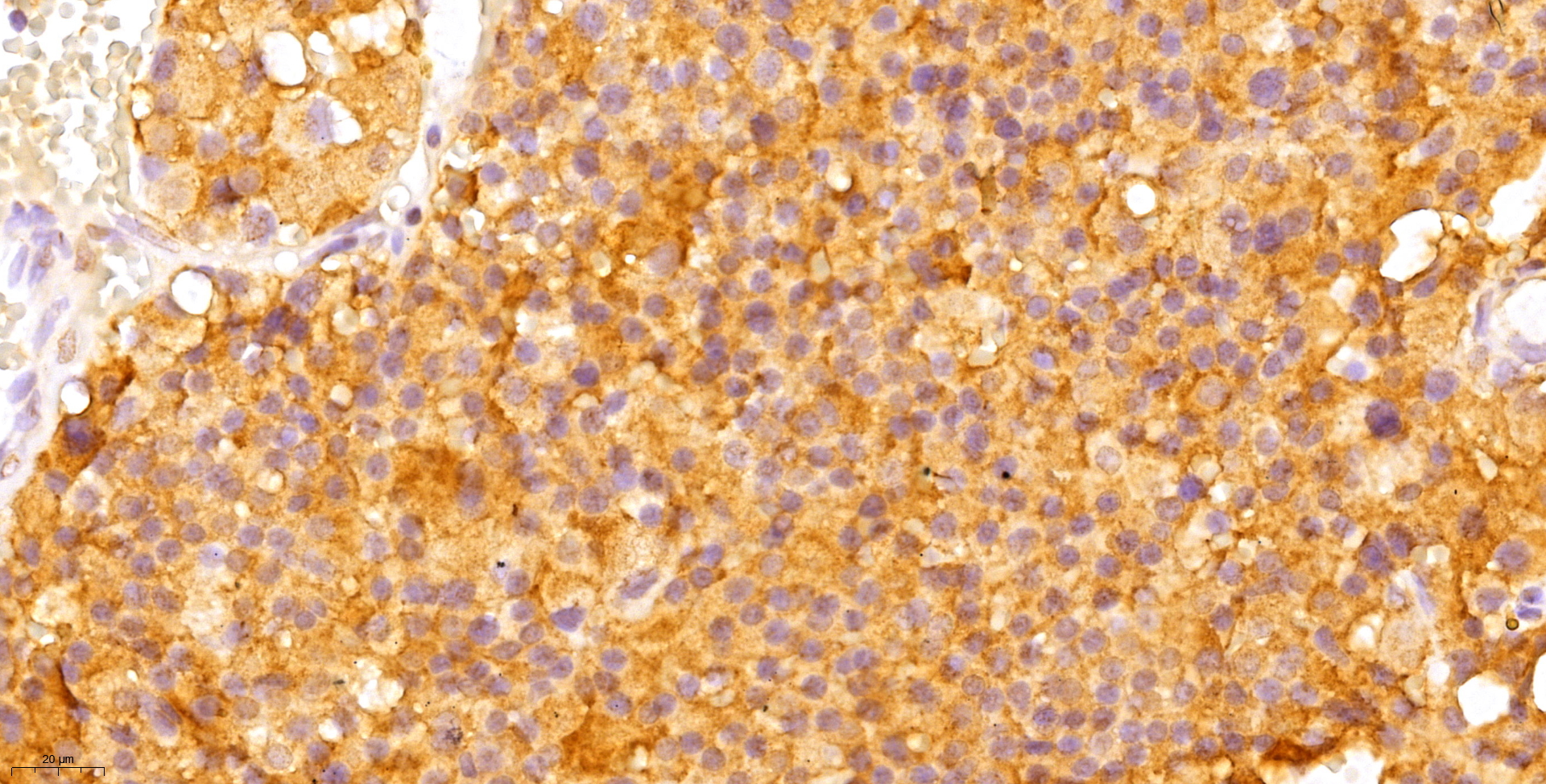

Supplement: Supplementary file 1 [file cells-15-01115-s001.zip › Supplementary Material/Figure2A.jpg]

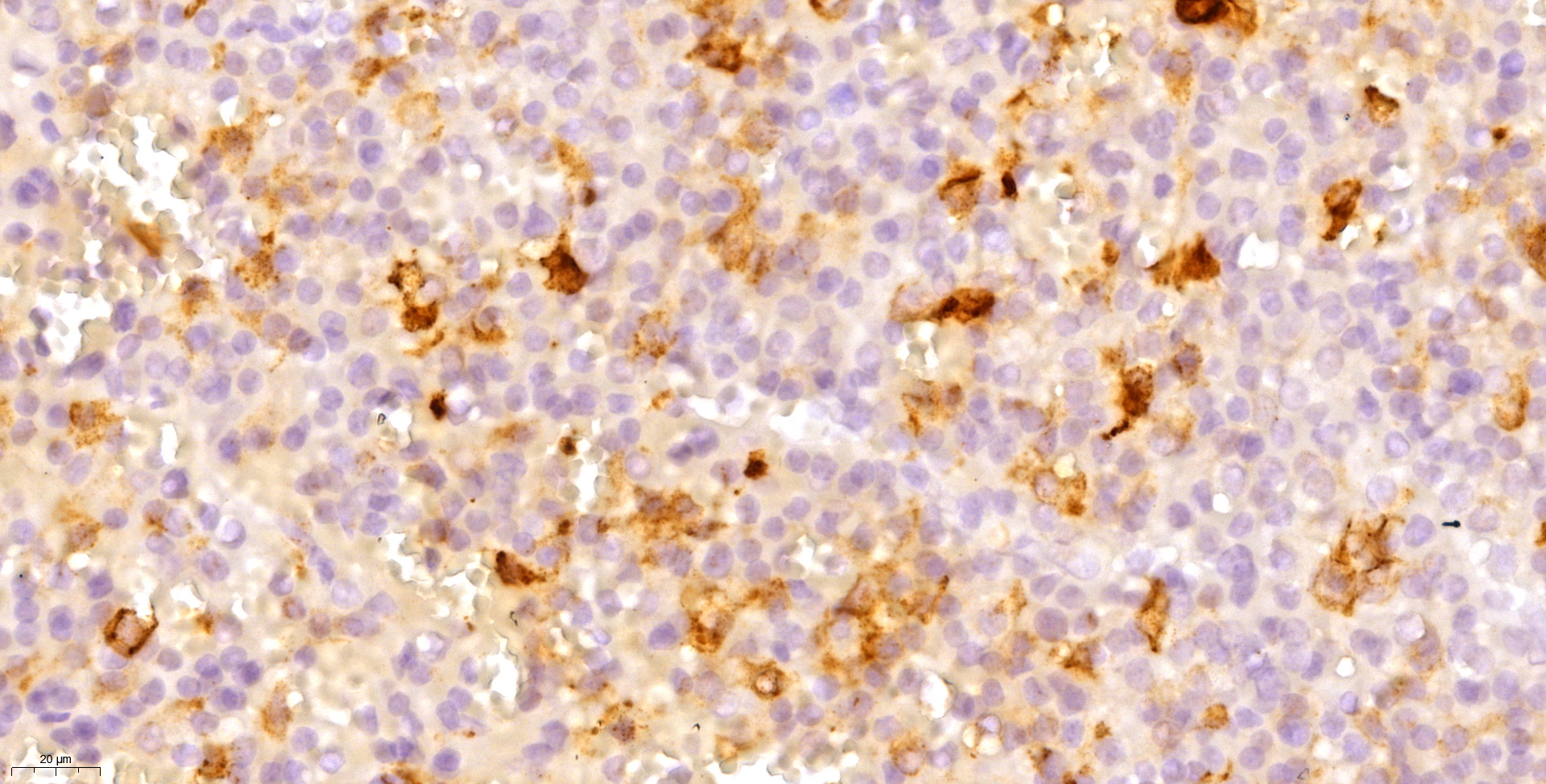

Supplement: Supplementary file 1 [file cells-15-01115-s001.zip › Supplementary Material/Figure2B.jpg]

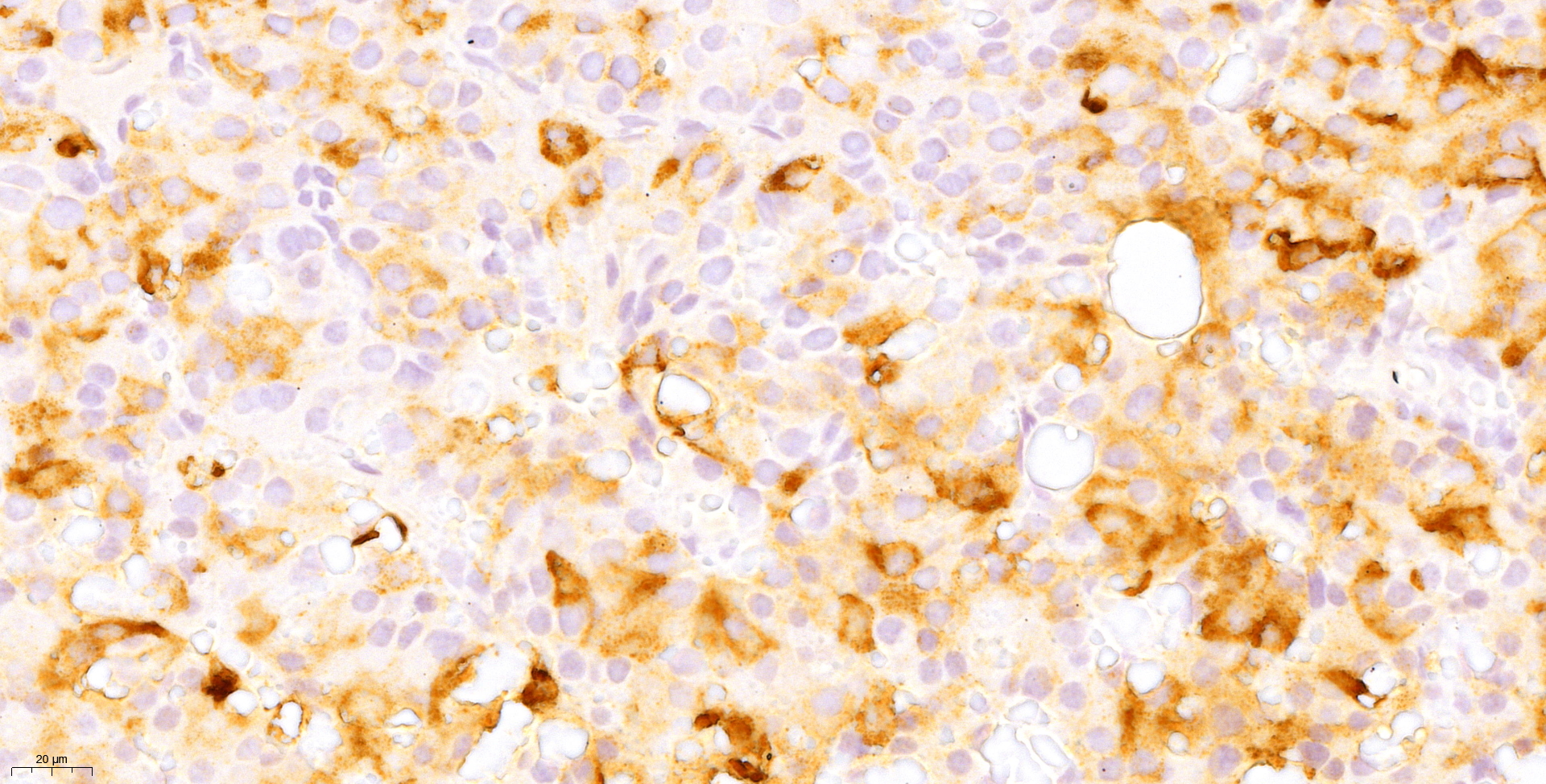

Supplement: Supplementary file 1 [file cells-15-01115-s001.zip › Supplementary Material/Figure2C.jpg]

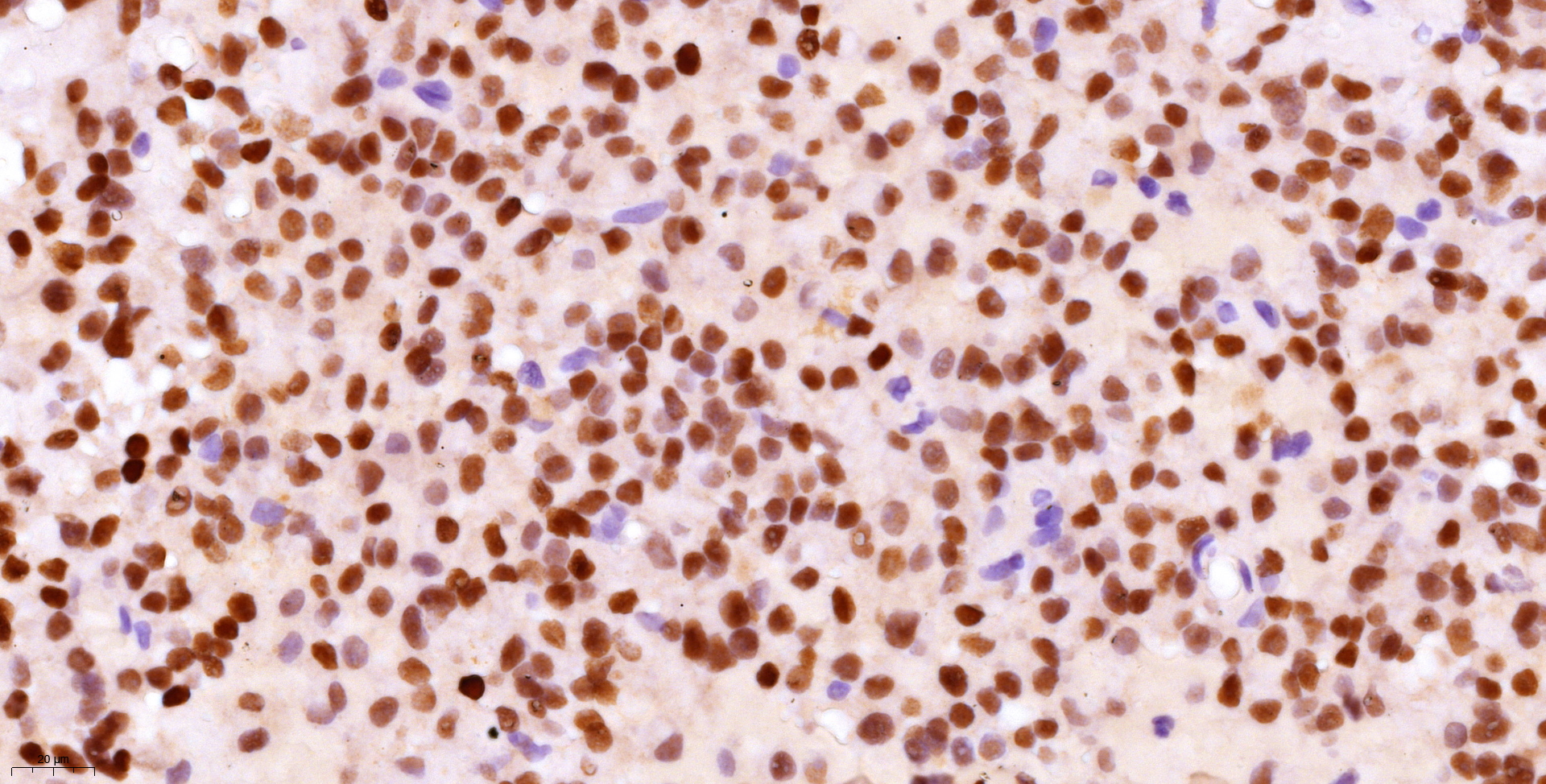

Supplement: Supplementary file 1 [file cells-15-01115-s001.zip › Supplementary Material/Figure2D.jpg]

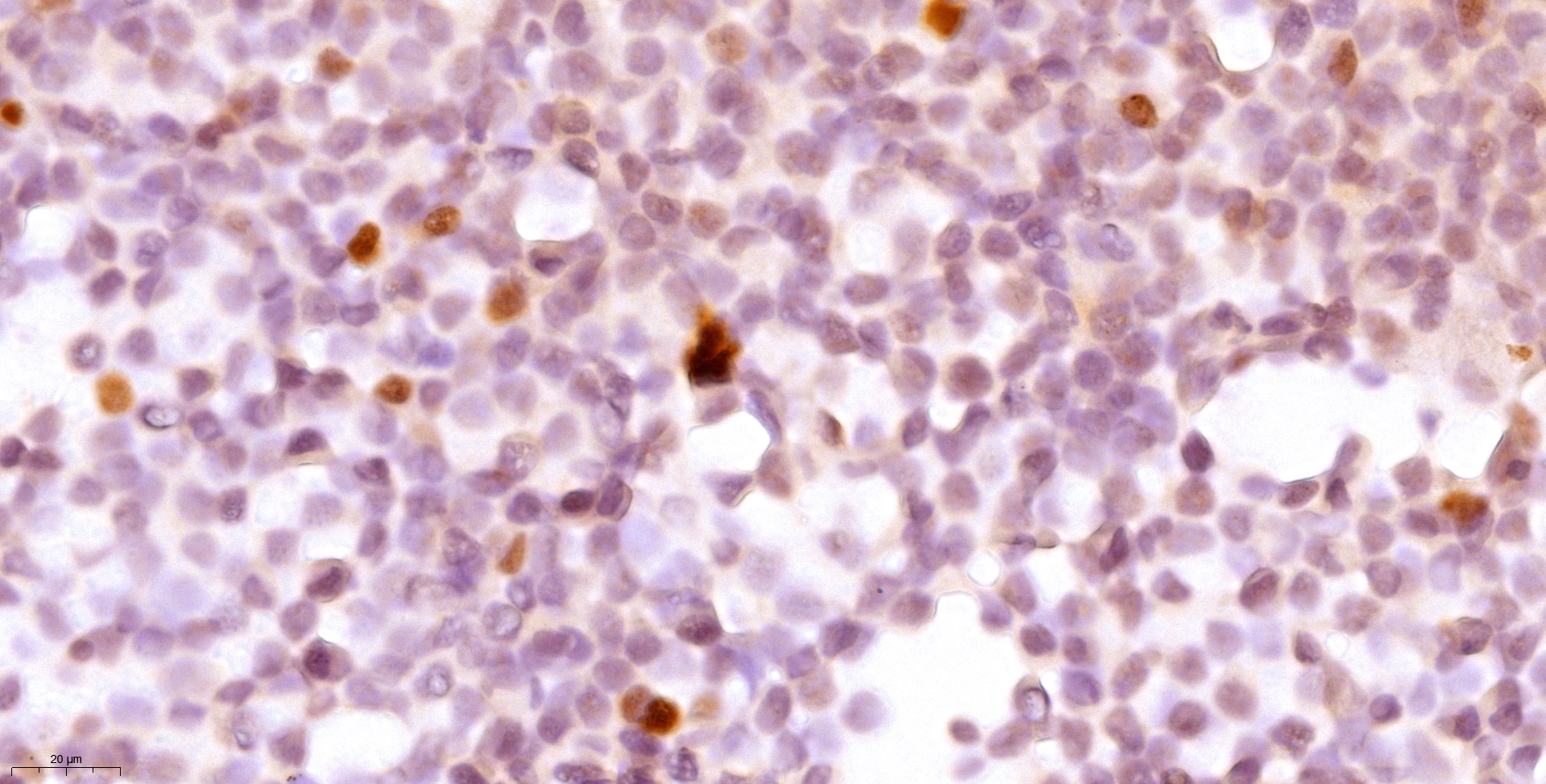

Supplement: Supplementary file 1 [file cells-15-01115-s001.zip › Supplementary Material/Figure2E.jpg]

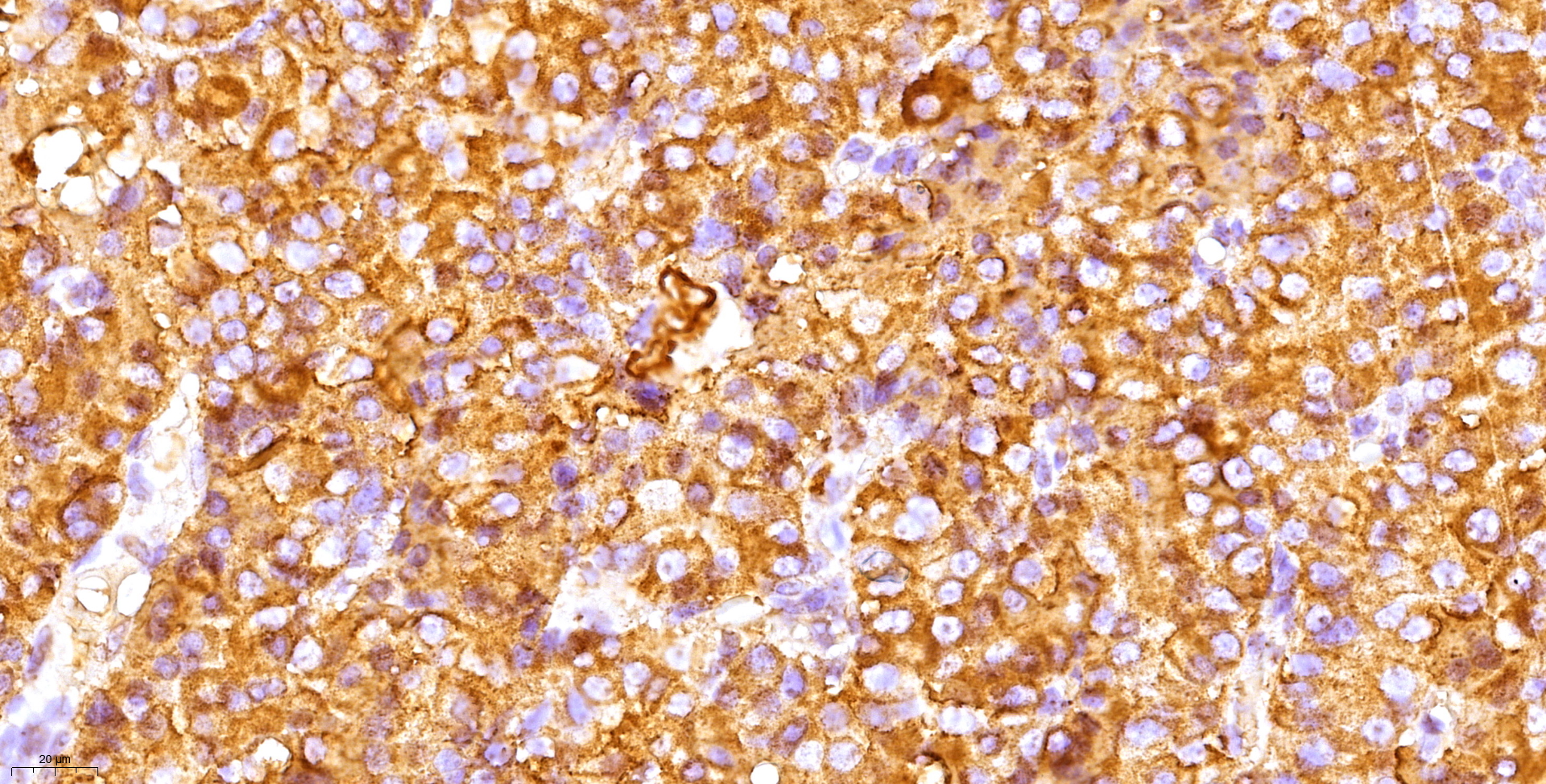

Supplement: Supplementary file 1 [file cells-15-01115-s001.zip › Supplementary Material/Figure3A.jpg]

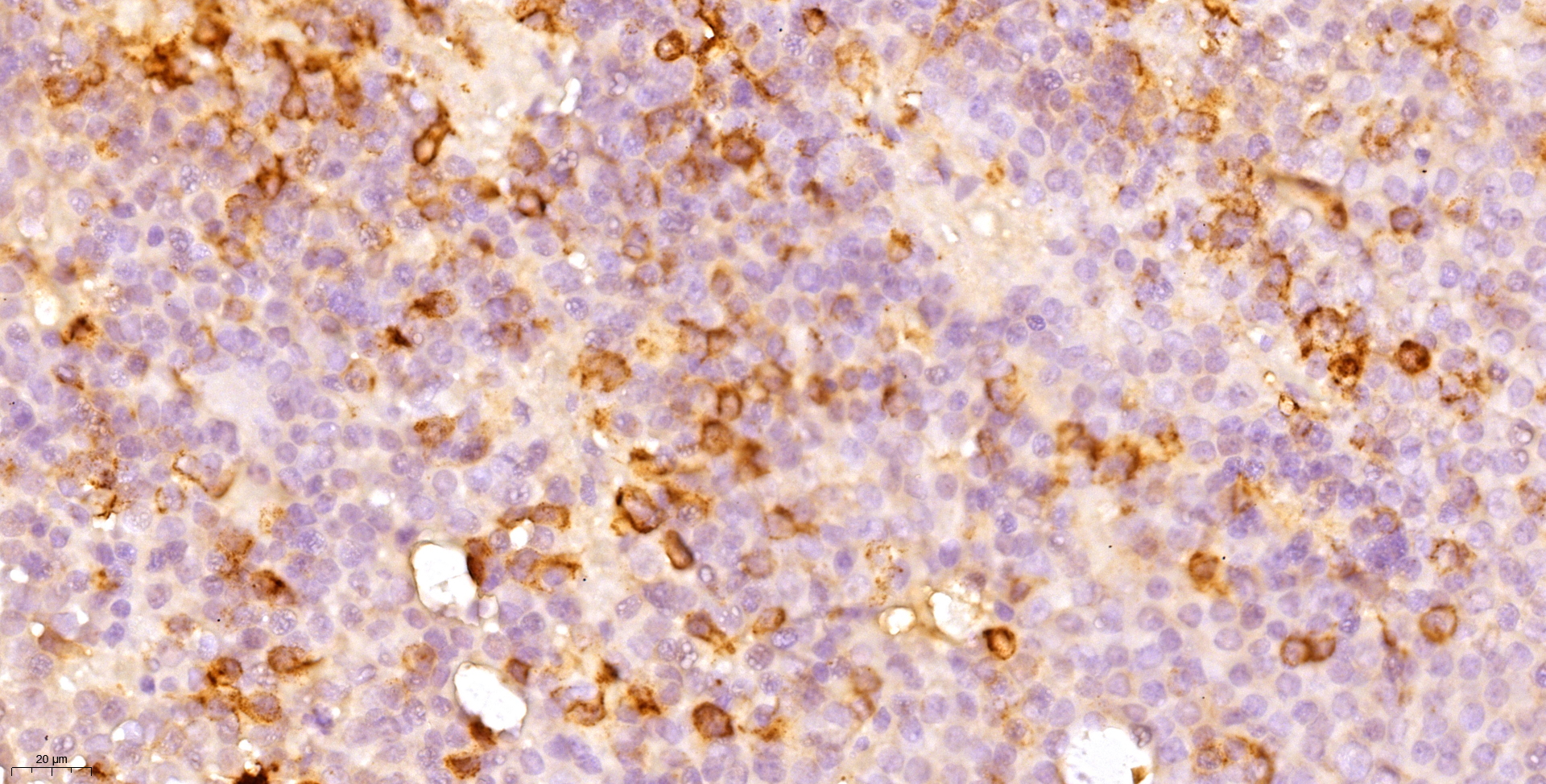

Supplement: Supplementary file 1 [file cells-15-01115-s001.zip › Supplementary Material/Figure3B.jpg]

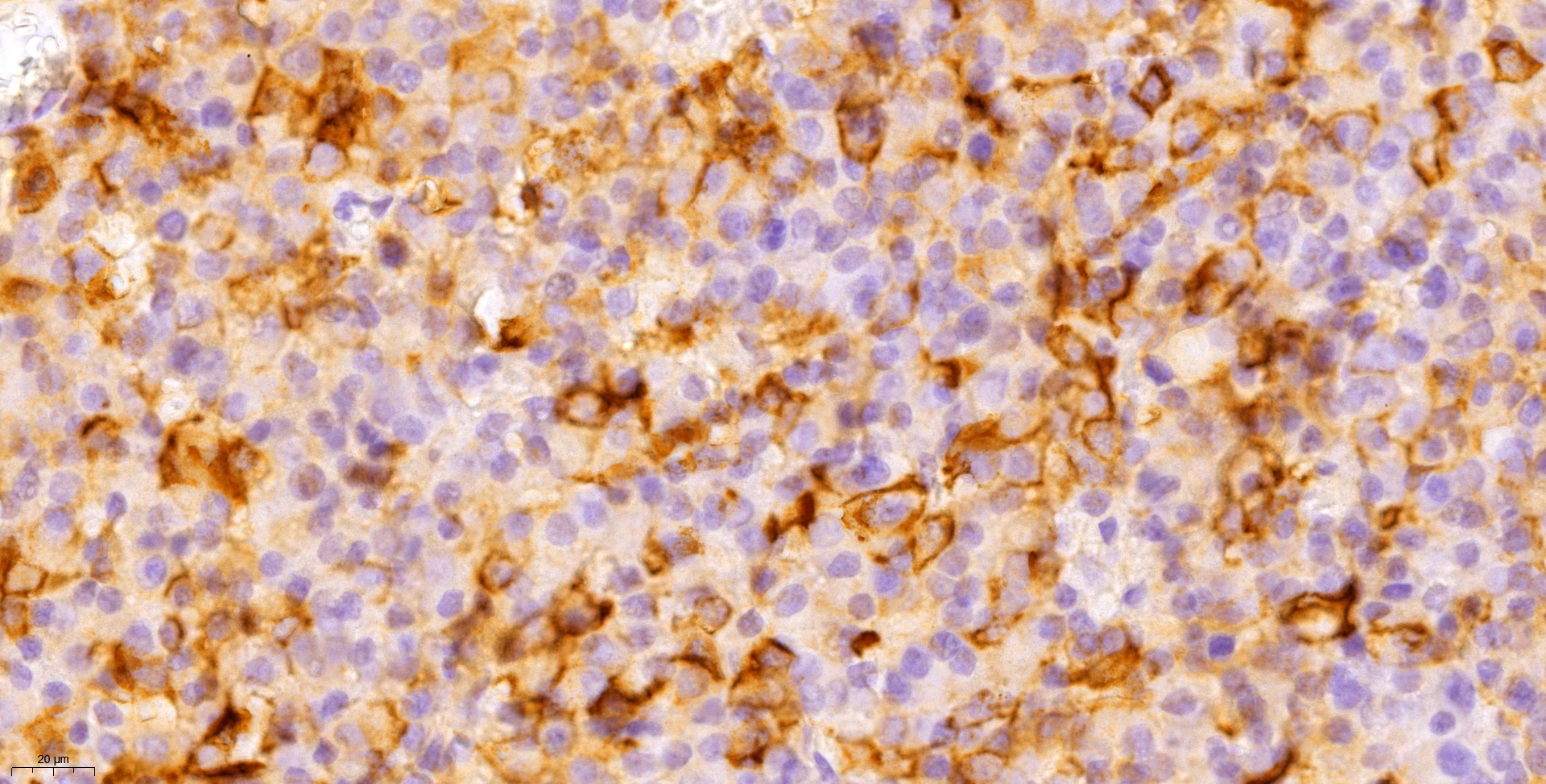

Supplement: Supplementary file 1 [file cells-15-01115-s001.zip › Supplementary Material/Figure3C.jpg]

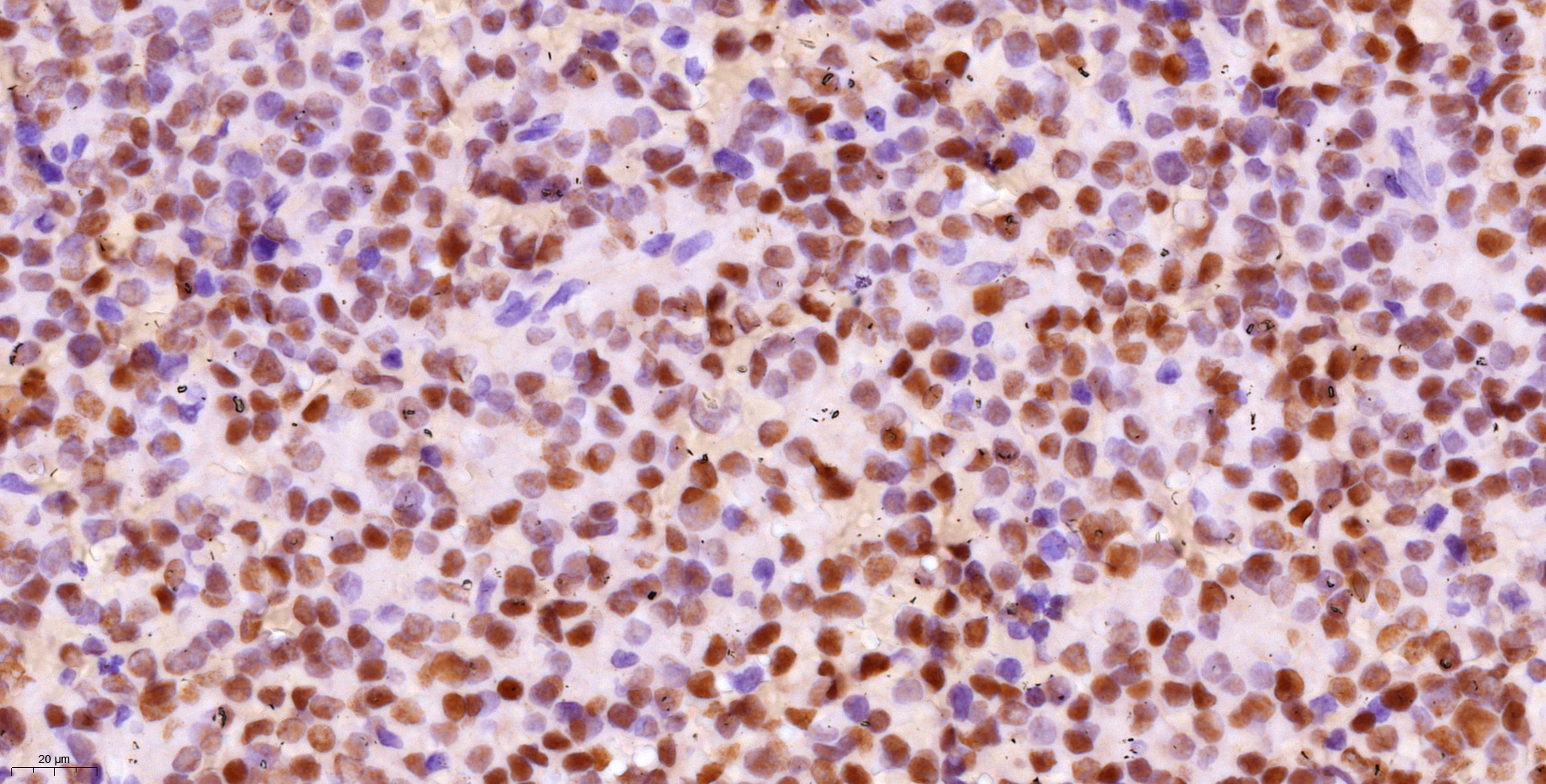

Supplement: Supplementary file 1 [file cells-15-01115-s001.zip › Supplementary Material/Figure3D.jpg]

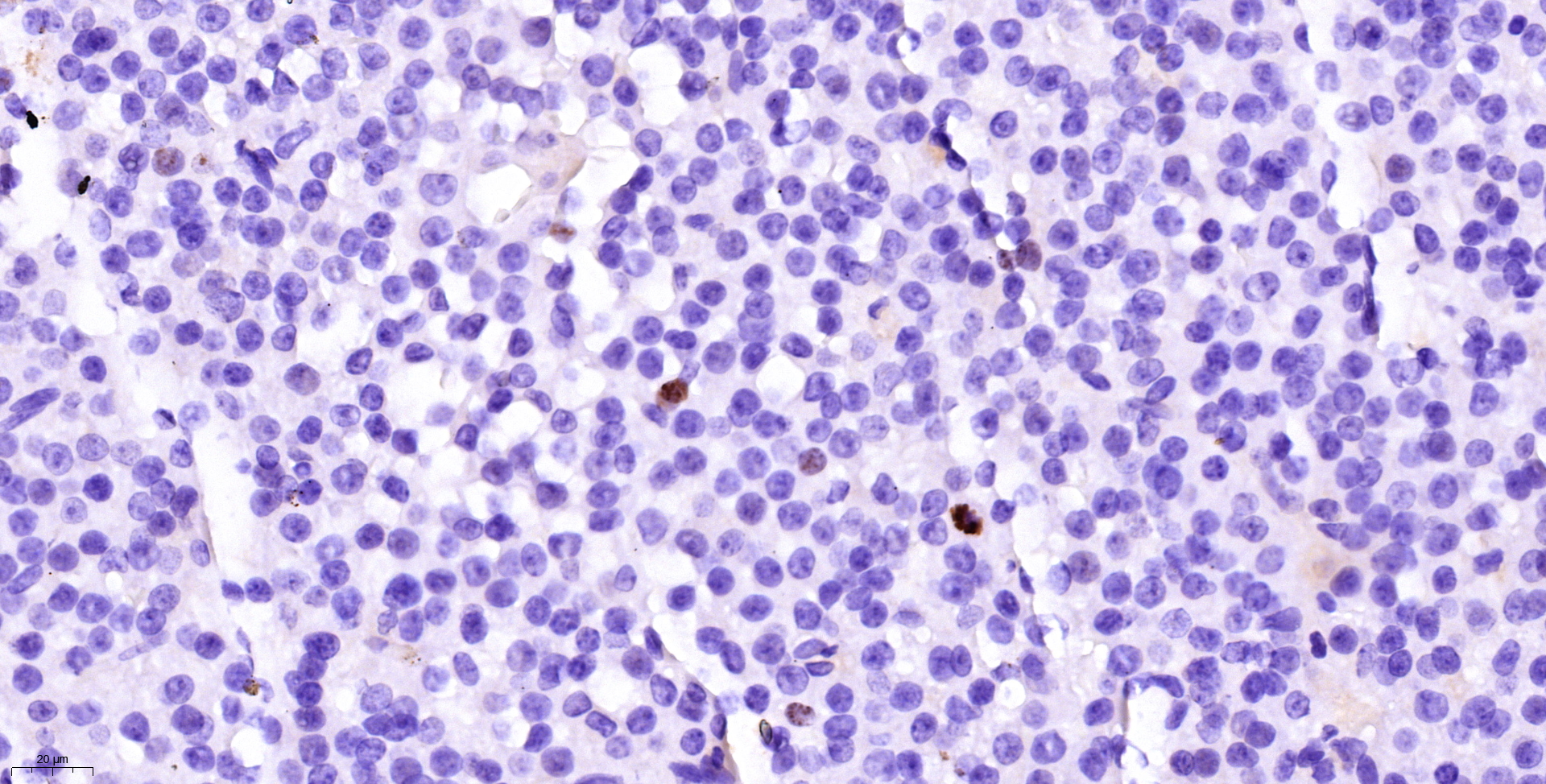

Supplement: Supplementary file 1 [file cells-15-01115-s001.zip › Supplementary Material/Figure3E.jpg]

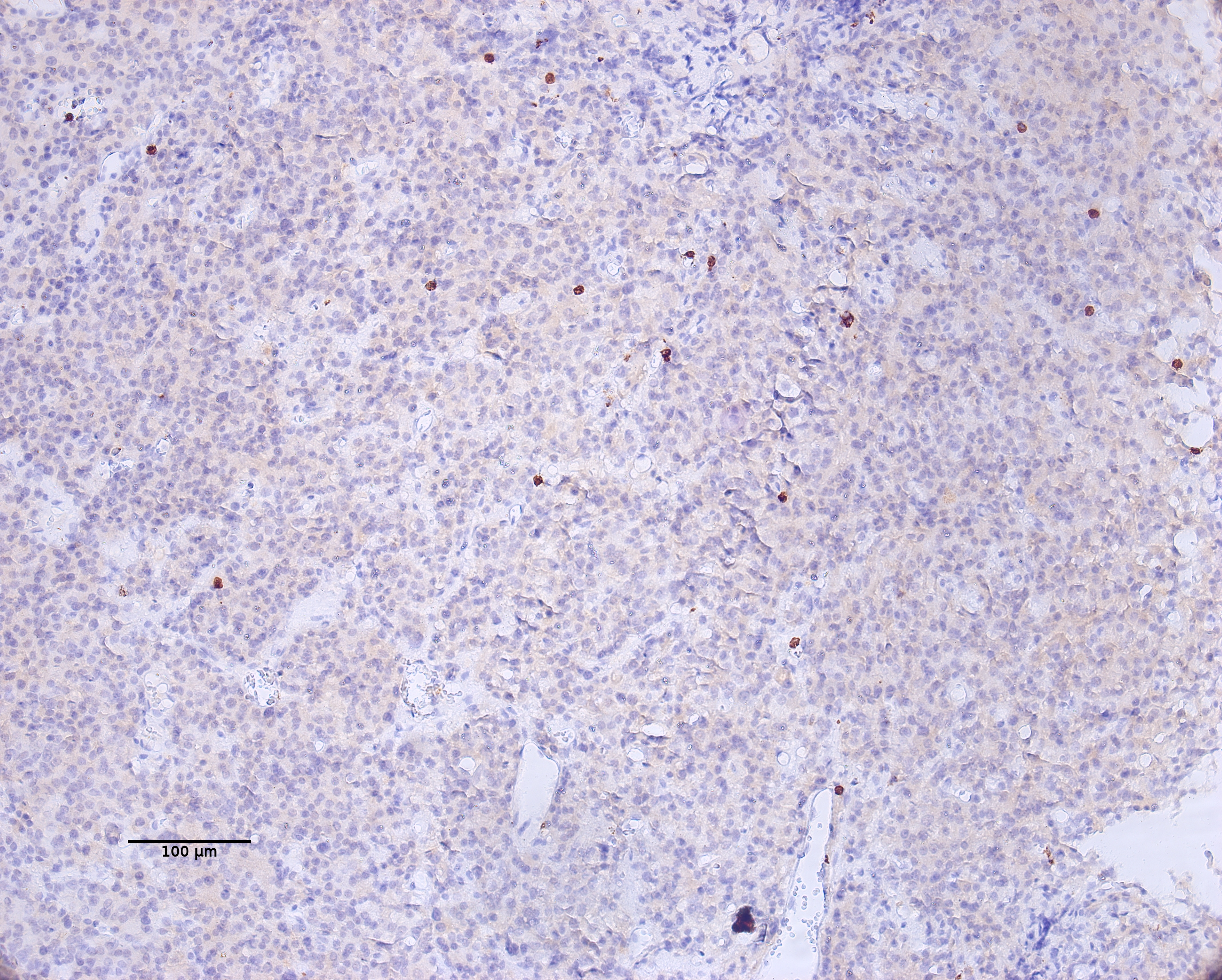

Supplement: Supplementary file 1 [file cells-15-01115-s001.zip › Supplementary Material/Figure4A.tif]

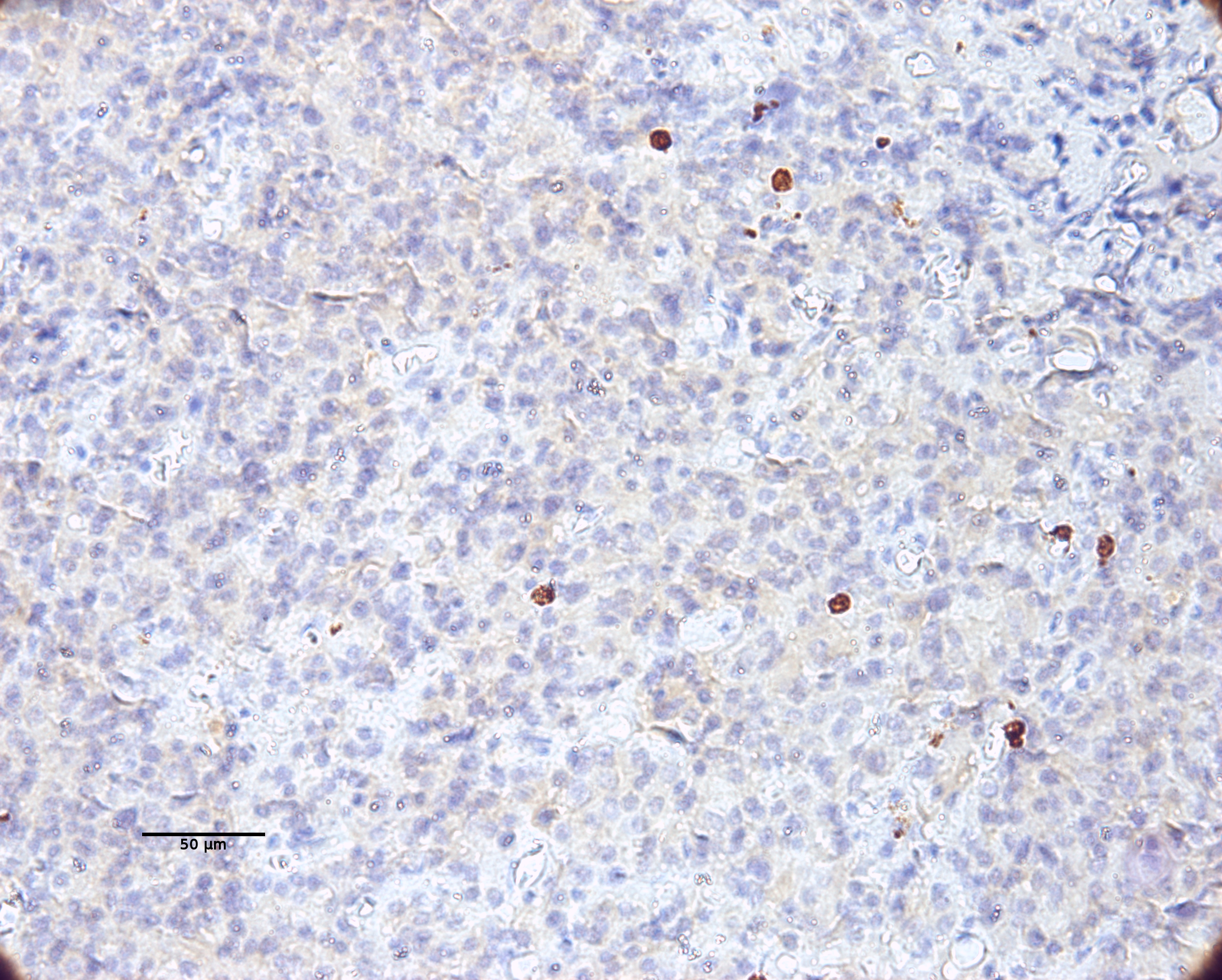

Supplement: Supplementary file 1 [file cells-15-01115-s001.zip › Supplementary Material/Figure4B.tif]

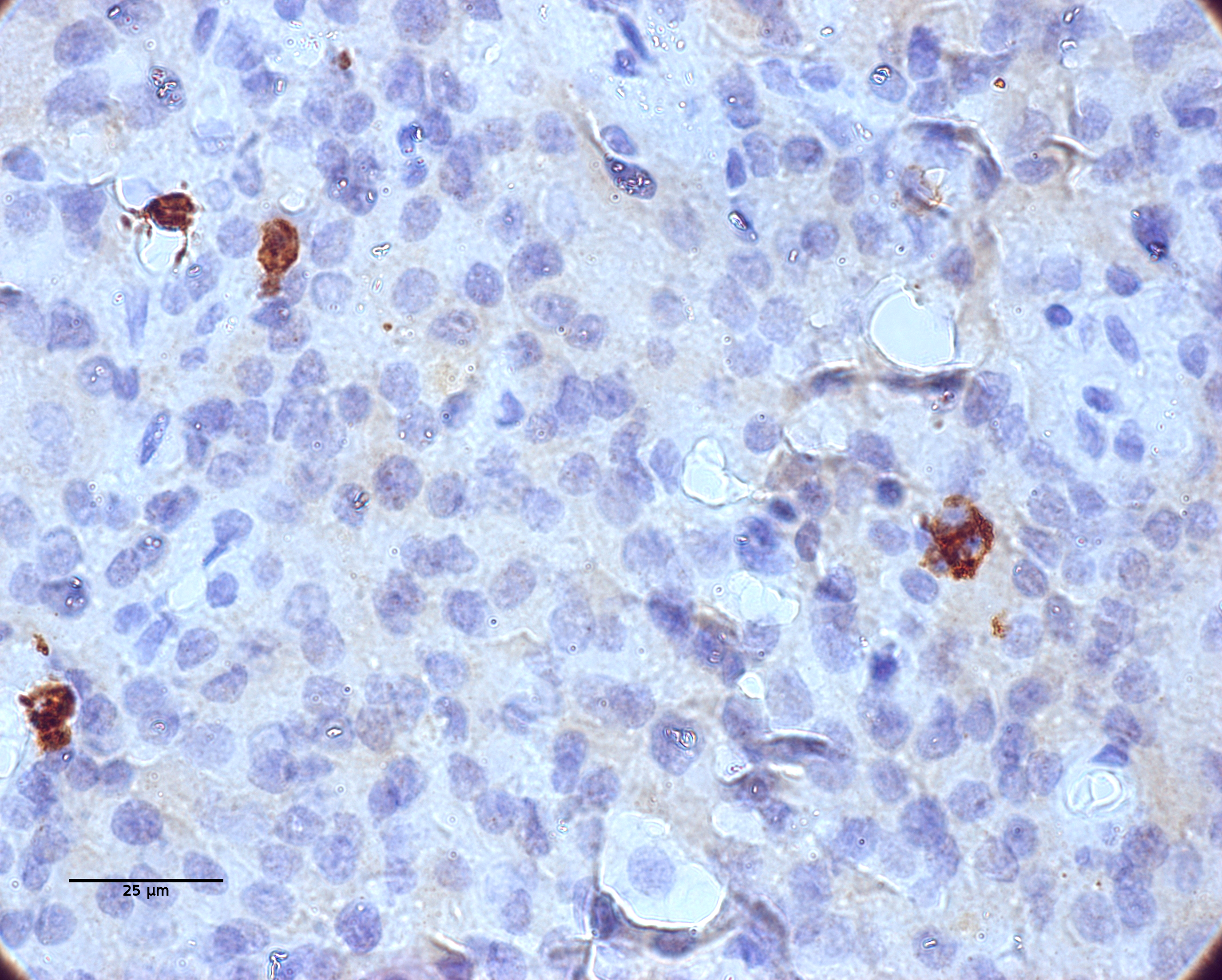

Supplement: Supplementary file 1 [file cells-15-01115-s001.zip › Supplementary Material/Figure4C.tif]

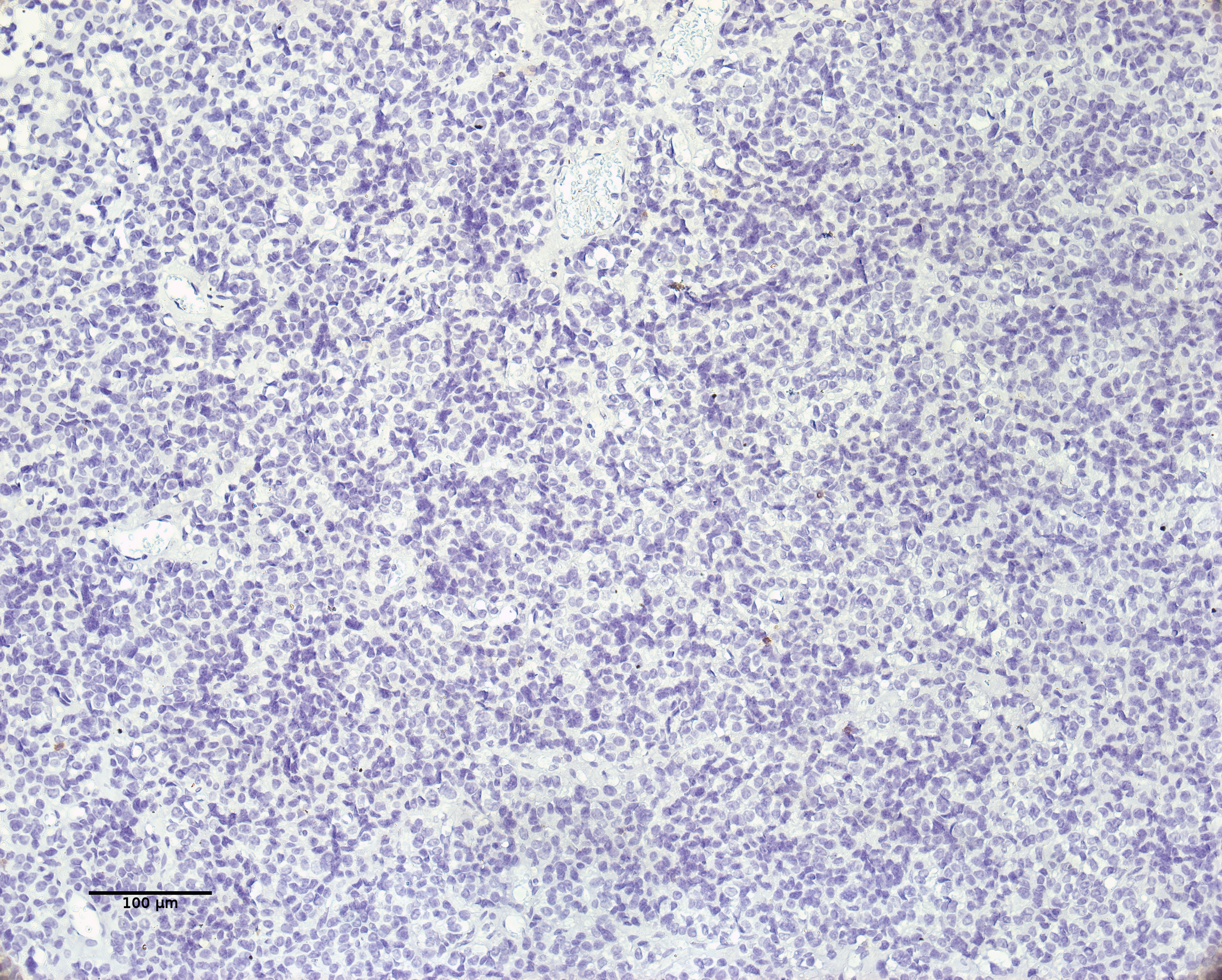

Supplement: Supplementary file 1 [file cells-15-01115-s001.zip › Supplementary Material/Figure4D.tif]

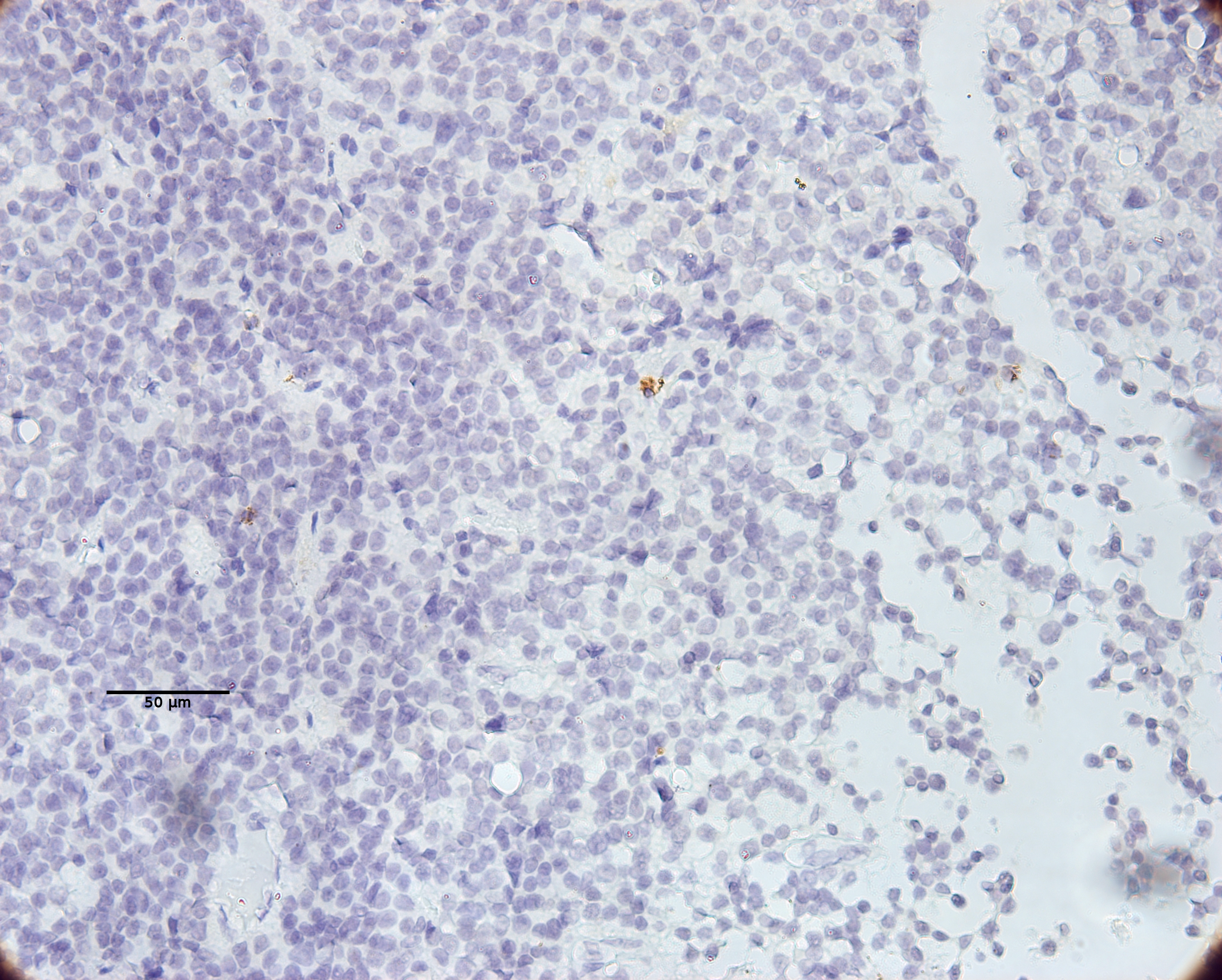

Supplement: Supplementary file 1 [file cells-15-01115-s001.zip › Supplementary Material/Figure4E.tif]

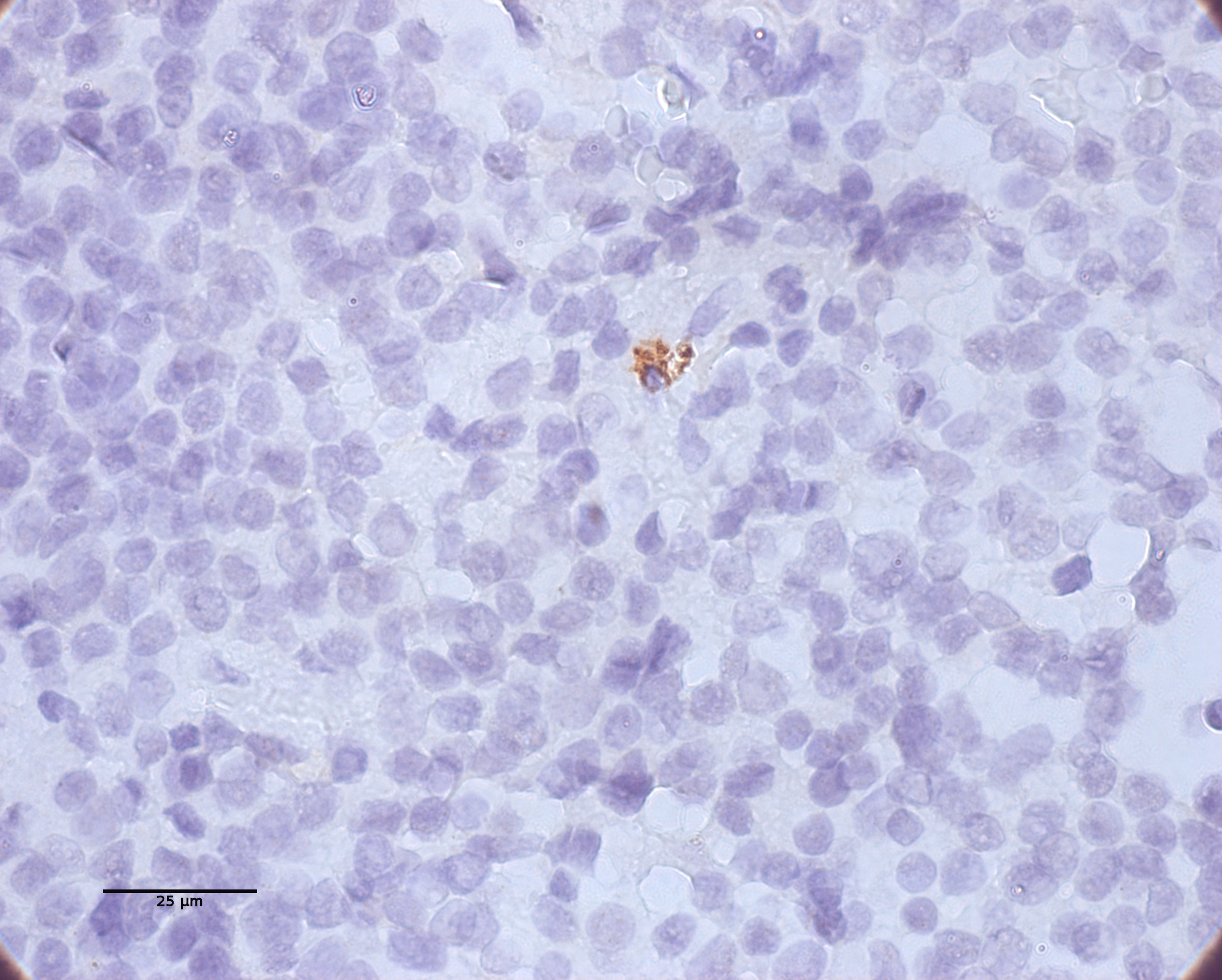

Supplement: Supplementary file 1 [file cells-15-01115-s001.zip › Supplementary Material/Figure4F.tif]
